# Well-defined porous membranes for robust omniphobic surfaces via microfluidic emulsion templating

**DOI:** 10.1038/ncomms15823

**Published:** 2017-06-12

**Authors:** Pingan Zhu, Tiantian Kong, Xin Tang, Liqiu Wang

**Affiliations:** 1 Department of Mechanical Engineering, The University of Hong Kong, Pokfulam Road, Hong Kong, China; 2 HKU-Zhejiang Institute of Research and Innovation (HKU-ZIRI), Hangzhou, Zhejiang 311300, China; 3 Guangdong Key Laboratory for Biomedical Measurements and Ultrasound Imaging, Department of Biomedical Engineering, Shenzhen University, 3688 Nanhai Avenue, Shenzhen 518060, China

## Abstract

Durability is a long-standing challenge in designing liquid-repellent surfaces. A high-performance omniphobic surface must robustly repel liquids, while maintaining mechanical/chemical stability. However, liquid repellency and mechanical durability are generally mutually exclusive properties for many omniphobic surfaces—improving one performance inevitably results in decreased performance in another. Here we report well-defined porous membranes for durable omniphobic surfaces inspired by the springtail cuticle. The omniphobicity is shown via an amphiphilic material micro-textured with re-entrant surface morphology; the mechanical durability arises from the interconnected microstructures. The innovative fabrication method—termed microfluidic emulsion templating—is facile, cost-effective, scalable and can precisely engineer the structural topographies. The robust omniphobic surface is expected to open up new avenues for diverse applications due to its mechanical and chemical robustness, transparency, reversible Cassie–Wenzel transition, transferability, flexibility and stretchability.

## Abstract (web summary)

Designing mechanically and chemically robust liquid-repellent surfaces remains a longstanding challenge. Here, Wang and colleagues report a microfluidic emulsion templating strategy to fabricate bioinspired omniphobic porous membranes with remarkable durability.

The lotus leaf repels water due to its textured surface and hydrophobic wax covering^1^; it has inspired tremendous advances in the development of artificial superhydrophobic surfaces that are potentially promising in various areas including self-cleaning, anti-corrosion, anti-icing, droplet manipulation and water–oil separation^1^. Nevertheless, lotus-leaf-inspired superhydrophobic surfaces are prone to contaminating by low-surface-tension liquids^2^,^3^ such as oils, surfactant solutions and organic solvents. Overhang or re-entrant structures are necessary to repel low-surface-tension liquids where liquids are suspended on a composite solid–liquid–air interface^4^,^5^,^6^,^7^. The solid/liquid contact area is greatly reduced to improve the repellency and lower the adhesion of liquids in the non-wetting Cassie state^8^.

However, textured surfaces with requisite physical structures contribute significantly to mechanical fragility, because the reduced contact area yields high contact pressures upon mechanical load^9^. In addition, the overhang part of the grooved sidewall develops a predetermined breaking point that makes the re-entrant structures more fragile^10^. As such, the arrays of extruded structures^4^,^6^,^7^,^11^,^12^,^13^,^14^ are easily disrupted under external abrasion^15^ and can even collapse from the capillary forces for nanoscale structures^16^. This greatly restricts practical applications of liquid-repellent surfaces. The mechanical durability has been improved by creating interconnected networks via reverse imprint lithography^17^, calcination^18^, colloid templating^19^,^20^ and the paint-adhesive method^21^,^22^,^23^, but these techniques have trade-offs in terms of liquid repellency^17^,^20^, regularity of surface architectures^18^,^21^,^22^,^23^ and/or transparency^21^,^22^,^23^. New structural designs are needed to mutually optimize the liquid repellency and mechanical durability, as well as transparency, transferability and stretchability.

Springtails evolved a mechanically robust omniphobic cuticle. The cuticle is textured with uniform rhombic or hexagonal comb-like patterns of cavities where each cavity is enclosed by interconnected ridges and granules^10^,^17^,^24^,^25^,^26^. The re-entrant profiles of ridges and granules provoke the pinning of the three-phase contact line when springtails make contact with a liquid^24^. As such, an energetic barrier is developed to stabilize the non-wetting Cassie state with air trapped inside the cavities. Shiny air cushions are observed when springtails^24^ and their cuticle-replica^26^ are immersed into water^24^,^26^,^27^, oil^27^ and even ethanol^24^, indicating the omniphobicity. Furthermore, the structural feature of interconnected ridges and granules endows the cuticle with a higher mechanical stability^17^,^24^. Therefore, the springtail skin offers an exciting biomimicry opportunity as a blueprint for designing omniphobic surfaces with robustness in both liquid repellency and mechanical durability^10^.

Inspired by the springtail cuticle, we describe omniphobic surfaces combining excellent liquid repellency and remarkable durability using a bottom-up microfluidic emulsion templating (MET) technique. The resulting porous membranes have interconnected solid structures and uniform honeycomb-like micro-cavities with narrow openings that satisfy the re-entrant profiles. The solid/liquid contact area is precisely controlled via the membrane pore size. Moreover, this textured surface exhibits optimal stability in the non-wetting state. The robust omniphobicity was demonstrated by texturing an intrinsically amphiphilic polymer without any surface chemistry modification. Our approach is simple, low-cost, scalable and compatible with various transparent and flexible substrates. The resulting omniphobic surfaces are mechanically and chemically durable. This paves the way for new applications with high commercialization potential.

## Results

### Omniphobic surfaces by MET

**Figure 1:**
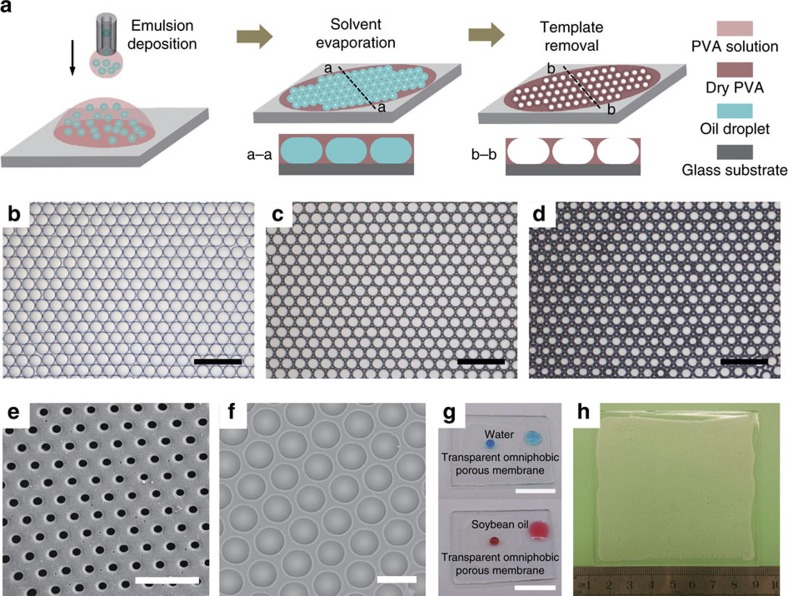
Fabrication of porous membranes by MET method. (**a**) Schematic of the fabrication process: emulsion generation, emulsion deposition, solvent evaporation, and template removal. The emulsion generation and emulsion deposition can be performed either simultaneously or independently. (**b**) Optical micrograph showing the self-assembly of micro-droplets into dense-packed hexagonal arrays after emulsion deposition. (**c**) Optical micrograph of the dry PVA membrane embedded with oil templates after solvent (water) evaporation. (**d**) Optical micrograph of the porous PVA membrane after template removal. (**e**,**f**) Scanning electron microscope (SEM) images of the porous PVA membrane with the uniform opening of different sizes. (**g**) Transparency of the porous PVA membrane. Both water (top, dyed with methylene blue) and soybean oil (down, dyed with oil red O) droplets bead up on the porous part (left) but are more wettable on the smooth part of the membrane (right). (**h**) Wafer-scale fabrication of the porous membrane on an 8 × 8 cm glass substrate by the MET method. Scale bars are 500 μm in **b**–**d**, 100 μm in **e**,**f** and 10 mm in **g**.

The MET fabrication process is shown schematically in Fig. 1a. Using the capillary microfluidic devices described in our previous study^28^,^29^,^30^,^31^, we first produced highly uniform emulsions where silicone oil droplets were dispensed in a polyvinyl alcohol (PVA) aqueous solution (Supplementary Fig. 1). The emulsion was then deposited onto a glass substrate. Within a few minutes, the lighter oil droplets floated to the air-water interface and became close-packed hexagonal monolayer arrays (Fig. 1b) so as to minimize the free energy of the system. Then the spherical oil droplets gradually deformed into pancake-like shape (a–a cross-section profile in Fig. 1a), owing to water evaporation and the resultant volume reduction in the PVA phase. The PVA later crystallized into dense-packed honeycomb structure when the water was completely evaporated (Fig. 1c). After removal of oil droplets, a porous membrane with micro-cavity structures was fabricated (Fig. 1d) and a narrow opening was created at the central top of the respective micro-cavity (b–b cross-section in Fig. 1a and Supplementary Fig. 2). The size of the uniform openings coincided with the top flat interface of the pancake droplet. This was well-controlled by the PVA concentration (Fig. 1e,f and Supplementary Fig. 2). Moreover, the fabricated polymer membrane is transparent (Fig. 1g), because the absence of any sub-micrometer structures avoids the scattering of visible light (see Supplementary Fig. 3 for the ultraviolet-visible transmittance spectra). The MET method can be easily scaled up, thereby fostering the mass production of porous membranes for industrial applications. As a proof of concept, a wafer-scale fabrication of PVA porous membrane on an 8 × 8 cm glass substrate is shown in Fig. 1h.

**Figure 2:**
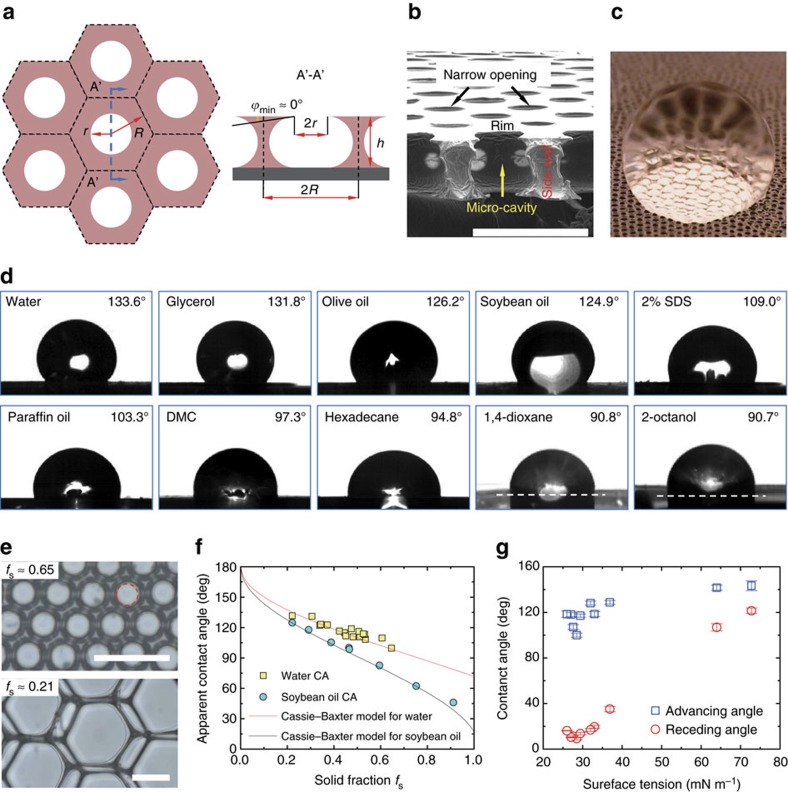
Omniphobicity of the porous membrane. (**a**) Schematic of the porous membrane from a top view (left) and a cross-sectional view (right). *R* is the radius of the incircle of the hexagonal cell, *r* is the opening radius, *h* is the height of the membrane and *ϕ*_min_ is the local minimum geometric angle of the sidewall profile. (**b**) Scanning electron microscope (SEM) image showing the cross-section of the PVA porous membrane. A re-entrant structure of the side wall is obvious. (**c**) Photograph of a water droplet suspended on the porous membrane in the Cassie state. The composite solid–liquid–vapour interface is manifested by the highly reflective region beneath the droplet. (**d**) Apparent contact angles θ* of ten test liquids on the porous membrane: water, glycerol, olive oil, soybean oil, 2% sodium dodecyl sulfate (SDS), paraffin oil, dimethyl carbonate (DMC), hexadecane, 1,4-dioxane and 2-octanol. These liquids represent various types: polar, nonpolar, inorganic and organic. θ*>90° for all test liquids suggests the omniphobicity. (**e**) Optical micrographs of the porous membranes with different values of the solid fraction *f*_s_. The opening is guided by the dashed circle in the upper image. (**f**) Comparison between experimental results and Cassie–Baxter predictions for apparent contact angles of water and soybean oil on porous membranes. Each data is the average of three to five measurements depending on the size of the sample for the measurement. (**g**) Advancing and receding angles of liquids with various surface tensions on the porous membrane. Error bar stands for the s.d. of five measurements. Scale bars, 100 μm.

The synthetic porous membrane resembles the springtail cuticle in its comb-like cavity and interconnected polymer structures with re-entrant profiles (Fig. 2a,b). Re-entrant structures exist on sidewalls between adjacent cavities because the narrow opening (of radius *r*) is always smaller than the micro-cavity in size, as shown in the schematic (Fig. 2a) and the corresponding scanning electron microscope image (Fig. 2b). Figure 2b also displays a closed cell of each micro-cavity separated by the impermeable sidewalls, mimicking the enclosed cavity structure in springtail cuticle. The minimum geometric angle *ϕ*_min_ is situated at the rim of the opening with a value very close to 0° (Fig. 2a,b) due to the natural deformation of the oil droplet. The non-wetting Cassie state could be maintained for a liquid possessing an equilibrium contact angle *θ*_*Y*_ (contact angle on a smooth surface with identical surface chemistry) larger than *ϕ*_min_ (ref. 4). As *ϕ*_min_≈0° and *θ*_*Y*_≥0°, the Cassie state is expected. Therefore, we speculate that the porous membrane displays omniphobicity.

To confirm the speculation, we visualized a water droplet (*θ*_*Y*_=71.7±0.3° for water on smooth PVA) deposited on the crosslinked PVA porous membrane (crosslinked with glutaraldehyde (GA) to avoid dissolution) where a highly reflective area underneath the droplet reveals the formation of a composite solid-liquid-air interface denoting the Cassie state (Fig. 2c). When the PVA porous membrane was immersed in water and soybean oil, we also observed the shiny air cushion trapped in the micro-cavity structure (Supplementary Fig. 4), very much resembling the air plastron occurring on the immersed springtail body^24^,^27^. We then measured the apparent contact angle θ^*^ of ten liquids with various surface tensions on the PVA porous membrane—all of which are larger than 90° (Fig. 2d). These results demonstrate the omniphobicity of the micro-cavity-decorated PVA membrane without any surface chemistry modification. It is worth noting that PVA is intrinsically an amphiphilic material, and some of the test liquids readily wet the smooth PVA surface with *θ*_*Y*_<10° (see Supplementary Table 1 for *θ*_*Y*_ of all test liquids).

As the liquid droplet is in the Cassie state, the apparent contact angle θ* is predicted by the Cassie–Baxter model^8^:


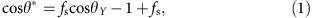


where *f*_s_ is the solid fraction at the top layer of the porous membrane. Considering the narrow opening with radius *r* and incircle of the hexagonal cell with radius *R* (Fig. 2a), we estimated *f*_s_ to be 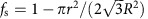. Consequently, *f*_s_ ranges theoretically from 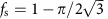 (≈0.093) to 1 with the lower and upper limit corresponding to *r*=*R* and *r*=0, respectively. By varying the oil droplet radius and PVA solution concentration, we could tune *r/R* and *f*_s_ across a wide range of values (Supplementary Fig. 2).

Figure 2e displays two surfaces with *f*_s_ of 0.65 and 0.21, respectively. The apparent contact angles for water and soybean oil on PVA membranes with different *f*_s_ agree well with equation (1) as indicated in Fig. 2f. Figure 2g displays the advancing and receding contact angles for ten liquids with surface tension *γ* ranging from 26 to 72.8 mN m^−1^. The contact angle hysteresis depends on surface tension^32^.

### Robustness of the re-entrant micro-cavity structure

**Figure 3:**
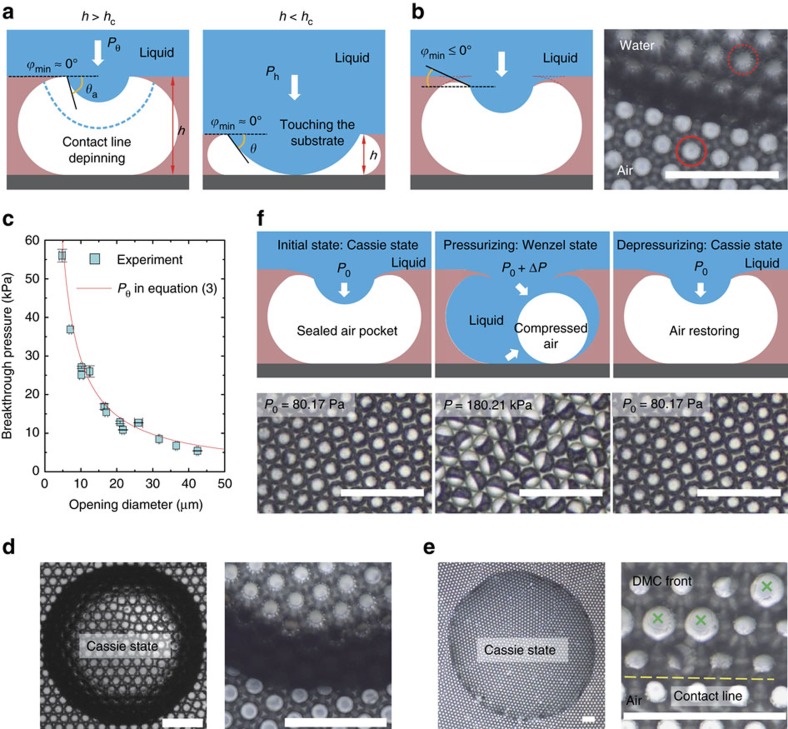
Cassie–Wenzel wetting transition. (**a**) There are two scenarios for the sagging liquid front. When *h*>*h*_c_, the three-phase contact line de-pins downward; when *h*>*h*_c_, the liquid front contacts the bottom substrate. Term *h*_c_ is the critical height of the membrane and is highly dependent on the opening radius, *r*, local minimum geometric angle, *ϕ*_min_, and liquid advancing angle, *θ*_a_. (**b**) Demonstration of the *ϕ*_min_≤0° upon liquid deposition, by which the rim of the opening is bent downward as contrasted by the dashed and solid circles in the lower image. (**c**) Experimental verification of the wetting transition occurring in the contact-line de-pinning scenario for the porous membrane—the MET method inherently endows the membrane with the property of *h*>*h*_*c*_. (**d**,**e**) Optical micrographs showing the persistence of the Cassie state for water (**d**) and DMC (**e**) during droplet evaporation. The right images in **d** and **e** are magnifications near the liquid front. Sparsely micro-cavities are wetted by DMC during evaporation (cross symbol marked), probably due to structure defects. (**f**) Schematic (upper row, cross-sectional view) and experimental observation (lower row, top view) of the reversible Cassie–Wenzel transition in a complete immersion test. Scale bars, 200 μm.

For a liquid with *θ*_*Y*_<90° beading up on micro-textured surfaces, the Cassie state is a metastable state that would irreversibly transition to the fully wetted Wenzel state^33^ when the transition barrier is overcome. Elevating the hydrostatic pressure in the liquid phase can induce the Cassie-Wenzel transition, for example, by droplet impact, droplet evaporation or increasing the depth of complete immersion in liquids. The liquid interface sags downward when the transition occurs. Depending on the height of the membrane, *h*, the sagging of the liquid front can occur through two transition scenarios^27^: contact line de-pins when *h* is larger than the critical height *h*_c_ (see the dashed liquid front in Fig. 3a, left schematic) or the liquid front can make contact with the substrate when *h*<*h*_c_ (Fig. 3a, right schematic). The breakthrough pressure *P*_break_ is defined as the critical pressure difference across the liquid-vapor interface for the transition and equals *P*_θ_ and *P*_h_ in the two scenarios, respectively. Theoretically, *P*_break_ is determined as follows (Supplementary Note 1 and Supplementary Fig. 5):


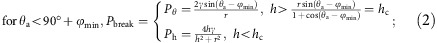



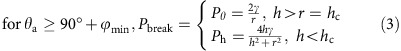


where *θ*_a_ is the advancing contact angle of the liquid on the smooth surface.

The breakthrough pressure indicates the stability of the Cassie state. A larger value of *P*_break_ denotes a more robust composite solid-liquid-vapor interface in the Cassie state. One should design the surface with *h*>*h*_c_ and *ϕ*_min_ as small as possible to render *P*_break_ higher, because *P*_θ_>*P*_h_ provided that *θ*_a_ is determined by the material's chemistry. The design of a re-entrant micro-cavity structure meets both criteria. First, the porous membrane created with the MET method intrinsically has 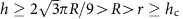 (Supplementary Note 2 and Supplementary Fig. 6). This yields *P*_break_ equal to *P*_θ_. Second, the flexible rim of the narrow opening is forced to bend downward by the elevated hydrostatic pressure upon liquid deposition. This gives *ϕ*_min_≤0° (Fig. 3b, for PVA material). For water, *θ*_a_=93.4±0.9°>90°+*ϕ*_min_ and *P*_break_ is then predicted by *P*_θ_ in equation (3). Figure 3c confirms this argument by showing good agreement between experimental measurements (see Methods) and theoretical predictions of *P*_break_. The large breakthrough pressure also indicates the persistence of the Cassie state during droplet evaporation as shown in Fig. 3d,e (also see Supplementary Note 3, Supplementary Fig. 7 and Supplementary Movies 1–3). In addition, the solid fraction *f*_s_ and breakthrough pressure *P*_break_ are decoupled for micro-cavity structures. This suggests the ability to achieve both high apparent contact angles and stable Cassie state by making *r/R* large, while *r* is small. This is readily realized by using a dilute PVA solution and small droplet templates in the MET method (Supplementary Fig. 2).

The re-entrant micro-cavities demonstrate a reversible Cassie–Wenzel transition, which has thus far only been achieved by hierarchical micro/nanostructures^26^,^34^ or by external stimuli^35^,^36^,^37^,^38^. The air pocket is sealed in a respective micro-cavity when the liquid deposit blankets the top openings (Fig. 3f, up left schematic), because the micro-cavity is enclosed and separated from each other (Fig. 2b). When the hydrostatic pressure exceeds the breakthrough pressure, the Cassie state transitions to the partial wetting state as the liquid gradually invades the micro-cavity and compresses the air pocket (Fig. 3f, up middle schematic). After releasing the elevated pressure, the compressed air expands and pushes the liquid out of the micro-cavity. The Cassie state then restores (Fig. 3f, up right schematic) when the applied pressure is lower than *P*_break_. We experimentally demonstrated this reversible process in Fig. 3f (lower images, see also Supplementary Movie 4). Initially, at 80.17 Pa the liquid (water) was in the Cassie state. Then the non-wetting Cassie state transitioned to the partial wetting state (*P*_break_≈16.8 kPa) when the pressure was controlled to gradually increase (see Methods) to 180.21 kPa, where every micro-cavity was in a Janus state (Fig. 3f, lower middle image): the light part indicates water invasion, while the dark part denotes the compressed air pocket (Supplementary Fig. 8). Then, the Cassie state recovered when the pressure was decreased back to 80.17 Pa. The reversible process was successfully observed for at least 5 cycles. It is noteworthy that the PVA omniphobic surface is structural stable under the hydrostatic pressure of at least hundreds of kPa, in comparison with the several kPa in mechanical stability for the abrasion test^39^.

### Mechanical properties of the porous omniphobic surface

**Figure 4:**
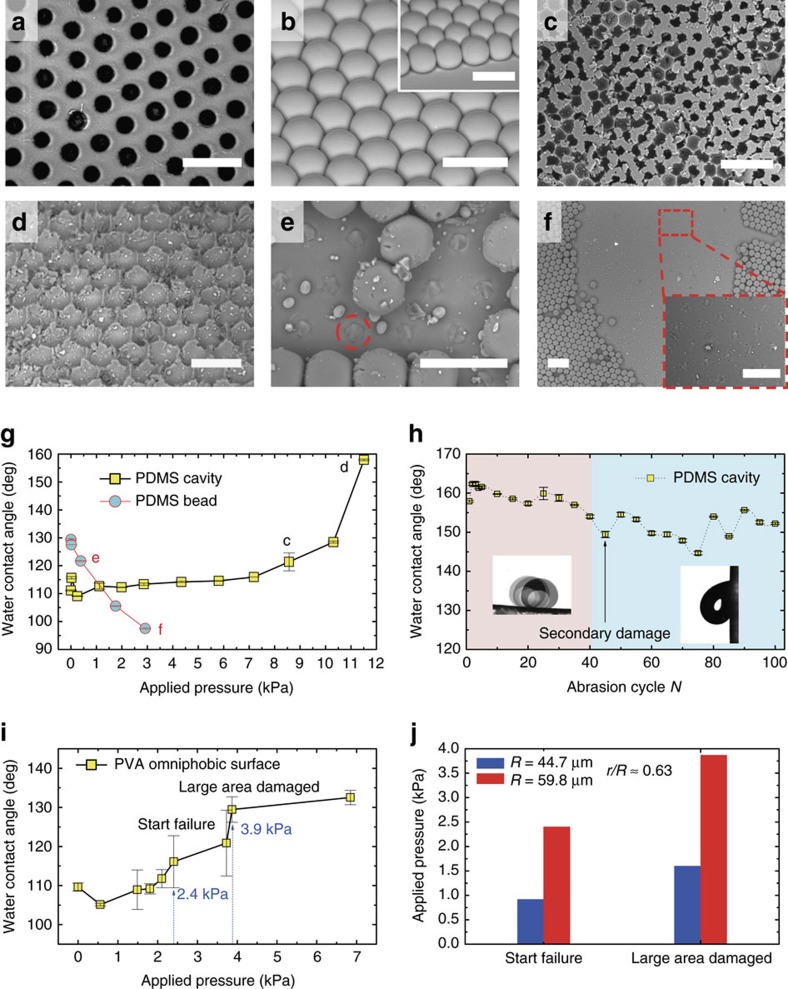
Improved mechanical stability of the interconnected micro-cavity structure. (**a**,**b**) Scanning electron microscope (SEM) images showing PDMS surfaces with interconnected micro-cavity (**a**) and discrete micro-bead (**b**) structures. (**c**,**d**) Structure failure of the top layer on PDMS micro-cavity surface under applied pressures of 8.6 kPa (**c**) and 11.5 kPa (**d**) in the sandpaper abrasion test. The integrity of the top layer starts to be damaged in **c** and is totally destroyed in **d**; the bottom micro-cavity structure is preserved. (**e**,**f**) Structure failure of the PDMS micro-bead surface under applied pressure of 0.4 kPa (**e**) and 2.9 kPa (**f**). The micro-bead structures start to fail at the bottom in **e** and large areas are damaged in **f**. (**g**) Apparent water contact angle versus applied pressure on PDMS micro-cavity and micro-bead surfaces. (**h**) Mechanical durability of the PDMS micro-cavity surface. As the top layer is abraded, the initial water contact angle is around 160°, corresponding to the last data in **g**. After 40 cycles of abrasion at an applied pressure of 11.5 kPa, a secondary damage of the bottom micro-cavity structure starts. (**i**) Apparent water contact angle versus applied pressure on PVA porous surface. The top-layer structure begins to fail at 2.4 kPa and a large area of the top layer is destroyed at 3.9 kPa. (**j**) Comparison of the critical destructive pressure for two PVA porous membrane with the same opening ratio (*r/R*≈0.63) but different size of the micro-cavity *R*. The increased critical pressure with *R* denotes a mechanically more stable structure for larger micro-cavity. Error bar stands for the s.d. of ten measurements. Scale bars are 100 μm in **a**,**b**,**d**,**e** and insets of **b**,**f**, and 200 μm in **c**,**f**.

The porous membranes with interconnected structures exhibit improved mechanical stability compared to surfaces with discrete structures such as pillars, nails and beads. We performed wearing tests (see Methods) to quantify the mechanical robustness of the surfaces where a loaded surface is abraded with a sandpaper^21^. To isolate the effect of structural topography, two polydimethylsiloxane (PDMS) surfaces with micro-cavity and micro-bead structures were fabricated from the same mould (see Methods) and tested (Fig. 4a,b, respectively). As the load was added, the increased friction (between sandpaper and test surface) gradually destroyed the structural integrity of the porous PDMS surface (Fig. 4c, 8.6 kPa) and then swept away the top layer of the porous surface leaving the bottom cavity structures. This increased the surface roughness (Fig. 4d, 11.5 kPa). In comparison, the micro-bead structures failed directly at the bottom (dashed circle in Fig.4e, 0.4 kPa) and a large area of the structures was completely removed due to the increased friction. This made the surface smoother as seen in Fig. 4f (2.9 kPa).

The water contact angle measurements indicate the mechanical robustness of the micro-cavity surface. Variations in water contact angle (Fig. 4g) denote the distinct structure-failure mechanisms of the two surfaces and highlight the distinguished mechanical stability of the interconnected porous structure. The critical pressure for micro-cavity failure (≈8.6 kPa, in Fig. 4c) is about 21.5 times higher than that of micro-bead (≈0.4 kPa, in Fig. 4e).

We further tested the durability of the same PDMS porous surface at a fixed pressure of 11.5 kPa after the top layer structure was damaged in Fig. 4d. In the first 40 abrasion cycles, the water contact angle is around 160° and the adhesion is quite small (left inset of Fig. 4h, sliding angle≈3.6°). After 40 cycle tests, the bottom structure (Fig. 4d) starts to fail as seen by the increased adhesion of water droplets (right inset of Fig. 4h, a sticky droplet on a 90° tilted surface) and large fluctuations in water contact angles (Fig. 4h). The structural failure of the bottom layer is secondary damage. The bottom structure is completely destroyed after 100 cycle abrasions (Supplementary Fig. 9). These results suggest that porous membranes with interconnected structures are mechanically stable and durable.

We also examined the mechanical stability of the PVA porous surface using the same sandpaper abrasion method. The structure-failure behavior is similar to that observed in PDMS porous surface, where the top layer structure is gradually ground away with the increased load but the bottom structure maintains (Supplementary Fig. 10). As the advancing angle of water on smooth PVA is *θ*_a_=93.4±0.9°, the water contact angle is expected to increase with applied pressure for the PVA omniphobic surface because of an increase in surface roughness (Fig. 4i). The critical pressure at structure failure is about 2.4 kPa for PVA porous surface, lower than PDMS porous surface, but still higher than the PDMS micro-bead surface. In addition, we found that the critical pressure increases with the micro-cavity size *R* when the opening ratio is constant (Fig. 4j), because the interconnected structures are wider and thicker for larger micro-cavities.

**Figure 5:**
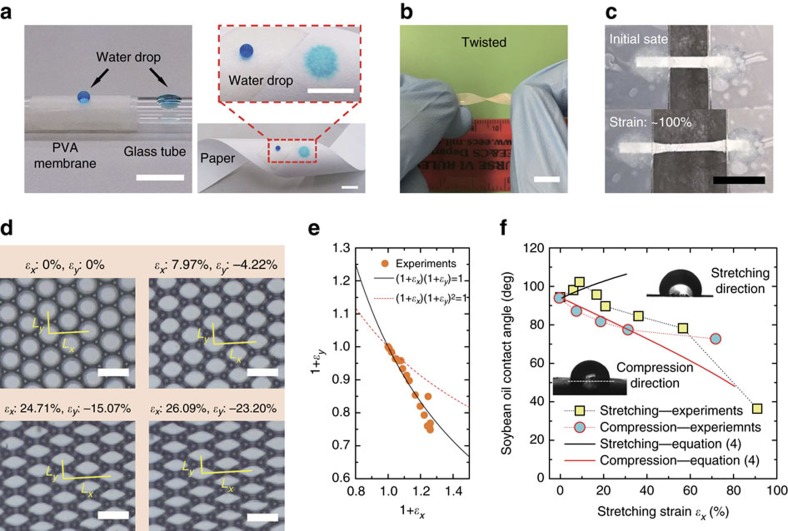
Flexibility of the porous omniphobic surface. (**a**) PVA porous membrane coated onto a cylindrical glass tube (left) and filter paper (right) using a double-faced adhesive tape. The water droplet (dyed with methylene blue) beads up on the coating part while readily wets the naked glass (left) and permeates through the filter paper (right), respectively. (**b**) Twisted PVA porous membrane. (**c**) Unidirectional stretching of the PVA porous membrane. (**d**) Microscopic deformation of the cavity structure under increasing stretching strain. Here, *ɛ*_*x*_ and *ɛ*_*y*_ stand for the stretching (*x* direction) and compression (*y* direction) strain, respectively. (**e**) Experimental validation of the relationship (1+*ɛ*_*x*_)(1+*ɛ*_*y*_)=1 for *ɛ*_*x*_<0.3. (**f**) Anisotropic apparent contact angles in the stretching and compression directions. Data is the average of five measurements. Scale bars are 10 mm in **a**–**c** and 100 μm in **d**.

The omniphobic surface is flexible made from soft materials such as PVA. The self-supporting surface can be readily transferred onto various materials with diverse shapes as an omniphobic coating, exemplified by a glass tube and a strip of flexible paper in Fig. 5a (see Supplementary Fig. 11 for other examples). The adhesion force between the omniphobic coating and the substrate can be quantified by a tensile test (Supplementary Fig. 12). The flexibility is also manifested in twisting (Fig. 5b) and stretching (Fig. 5c, Supplementary Fig. 13 and Supplementary Movie 5) of the porous membrane. When subjected to a stretch in the *x*-direction, the micro-cavities are compressed in the orthogonal *y* direction and deform gradually from circular into polygonal shapes (Fig. 5d and Supplementary Movie 6). The strain *ɛ* is defined as *ɛ*=(*L*−*L*_0_)/*L*_0_, with *L*_0_ and *L* denoting the initial and deformed dimensions of the surface, respectively. As such, *ɛ* is anisotropic in the *x* and *y* directions (*ɛ*_*x*_ and *ɛ*_*y*_, respectively, see Fig. 5d). For *ɛ*_*x*_<0.3, *ɛ*_*x*_ and *ɛ*_*y*_ are related to each other via (1+*ɛ*_*x*_)(1+*ɛ*_*y*_)=1 based on the unchanged surface area, instead of (1+*ɛ*_*x*_)(1+*ɛ*_*y*_)^2^=1 from the conservation of PVA volume (by assuming *ɛ*_*y*_=*ɛ*_*z*_), as shown in Fig. 5e. In fact, the top rim buckles at small tensile strain, by which the micro-cavity changes in its size but the film thickness keeps almost invariant (Supplementary Fig. 14).

The anisotropic feature of the deformed micro-cavity contributes to two distinct apparent contact angles along the stretching and compression directions (Fig. 5f). Based on the Cassie–Baxter model equation (1), the apparent contact angle on the deformed surface 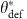 is predicted to be:


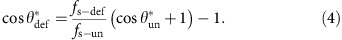


Here, 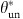 is the apparent contact angle on the undeformed surface, and *f*_s-def_ and *f*_s-un_ are solid fractions of the deformed and undeformed surfaces, respectively. We evaluated *f*_s-def_/*f*_s-un_=1/(1+*ɛ*_*x*_) in the stretching direction, and *f*_s-def_/*f*_s-un_=1/(1+*ɛ*_*y*_)=(1+*ɛ*_*x*_) in the compression direction (Supplementary Note 4 and Supplementary Fig. 14). The data show fairly good agreement with experiments at small strains (Fig. 5f). The decrease of 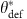 in the stretching direction (for *ɛ*_*x*_>10%) arises from the transition to the Wenzel state as a consequence of the rim buckling under strain (Supplementary Fig. 14) and the increasing opening radius, which decreases *P*_break_. In the compression direction, the deviation of experimental results from equation (4) for *ɛ*_*x*_>30% probably results from transition to plastic deformation at large strains (breakdown of (1+*ɛ*_*x*_)(1+*ɛ*_*y*_)=1, see Supplementary Fig. 15).

### Chemical stability characterization

**Figure 6:**
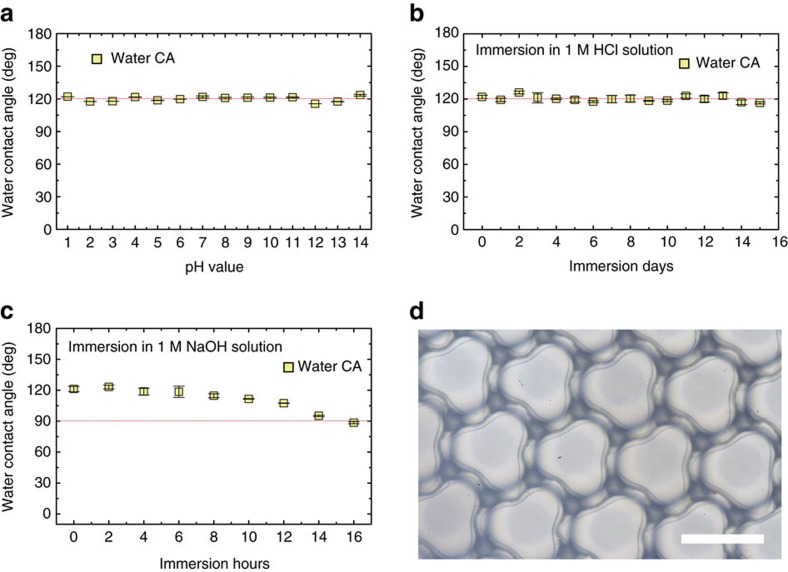
Chemical stability test. (**a**) The apparent contact angle of water droplets with pH values ranging from 1 to 14. The constant contact angle indicates a chemically stable structure with respect to acid and alkali. (**b**) The water contact angle versus days of immersion in 1 M HCl solution. The structure is excellently stable over 15 days. (**c**) The water contact angle versus hours of immersion in 1 M NaOH solution. The structure is stable in the first 8 h and then gradually deteriorated by NaOH. (**d**) The narrow opening deforms from a circular into a clover-like shape with rim buckling, losing the feature of re-entrant topography. Error bar stands for the s.d. of ten measurements. Scale bar, 100 μm.

Chemical stability is important in applications. As the PVA porous membrane displays omniphobicity in the absence of surface chemistry modification, the omniphobic surface is expected to be chemically stable unless the re-entrant structure is deteriorated by chemicals. Figure 6a shows the nearly constant contact angle of water droplets at various pH values, the re-entrant structure being thus chemically stable for both acid and alkali. The chemical durability has also been confirmed by immersing the crosslinked PVA omniphobic surface into 1 M HCl and 1 M NaOH solutions, respectively (Fig. 6b,c). The PVA membrane is superiorly stable for at least 15 days in HCl solution (Fig. 6b) and stable for 16 h in 1 M NaOH solution (Fig. 6c). The relatively lower chemical durability in alkaline solution is due to that the circular narrow opening transforms into the clover-like structure (Fig. 6d), partly wrecking the re-entrant profile necessary for omniphobicity.

## Discussion

In summary, we designed robust omniphobic surfaces with well-defined interconnected micro-cavity structures that were fabricated by a facile and cost-effective MET method. The fabrication process involves microfluidic emulsion generation, emulsion deposition, solvent evaporation and template removal. This method produces an omniphobic material from an intrinsically amphiphilic polymer without any surface chemistry modification. The omniphobicity originates from re-entrant structures on the narrow openings atop the micro-cavities. Varying the template size and precursor concentration enables independent control over the solid fraction and the opening radius such that the apparent contact angle and breakthrough pressure are decoupled. In addition, the fabrication method endows the omniphobic surface with two optimized geometric parameters: geometric angle *ϕ*_min_≈0° and structure height *h*>*h*_c_. These give the surface an optimal energetic barrier for the Cassie–Wenzel transition. Moreover, the MET method could be applicable to various materials including polymers, composites, inorganic oxides, metals and carbon that can dry out or be solidified^40^. Therefore, the MET approach could be an outstanding tool for the industrial-scale manufacture of omniphobic surfaces due to its facile, cost-effective and scalable nature.

Mimicking the springtail cuticle, the present omniphobic surface is featured by the ordered interconnected structures, tremendously distinct from surfaces with random or individually discrete structures. As such, the wetting process on the interconnected porous surface is different, for example, the reversible wetting transition observed in this study. The further insight into the wetting phenomena on the porous surface is yield by the generalized breakthrough pressure for the Cassie–Wenzel transition and the criterion for the persistence of the Cassie state during droplet evaporation (see Supplementary equations (8) and (9)). They are also applicable to other porous structures, such as hole-like and crater-like micro/nanostructures.

Besides, we envision far-reaching applications of the present omniphobic surface because it offers an unprecedented combination of multiple functions. First of all, the membrane with uniform porous structure can find applications in the design of novel gating systems^41^,^42^. Second, the omniphobic surfaces are applicable to solar panels, windshields and touch screens due to their transparency. The reversible Cassie–Wenzel transition would then benefit applications involving liquid immersion such as anti-fouling and drag reduction for fluidic systems and ship hull coatings. The sustained Cassie state during droplet evaporation makes the omniphobic surfaces suitable for surface patterning in various materials^43^ and biosensing^44^. Moreover, the enclosed micro-cavity prevents sideways propagation of the wetting transition, which is quite useful in isolating localized damages and defects of the surface. Furthermore, the free-standing surface can be transferred onto diverse materials, and its flexibility and stretchability may offer new opportunities in the design of flexible electronics for wearable^45^ and energy-storage devices^46^. The anisotropic wettability of the stretched surface is advantageous to directional liquid transportation^47^,^48^,^49^ such as for water collection^49^,^50^. More importantly, the robust mechanical and chemical stability of the surfaces would improve their longevity and preserve the omniphobicity in extreme environments. The multi-functionality of the omniphobic surfaces dramatically expands their spectrum of practical applications.

## Methods

### Fabrication of PVA porous membrane

The monodisperse emulsion was produced in capillary microfluidic devices. The dispersed phase was silicone oil (20 cSt; Aldrich) containing 2 wt% Dow Corning 749 Fluid (Dow Corning) as a surfactant, whereas the continuous phase was PVA (Mw 13,000–23,000, 87–89% hydrolysed; Aldrich) aqueous solution. The flow rate ratio of dispersed to continuous phase was varied to control the droplet size (Supplementary Fig. 1) so as to tune the size of the micro-cavities. To control the solid fraction, *f*_s_, the PVA solution was used from 2 wt% to 10 wt%. After generation, the oil-in-PVA emulsion was deposited onto a glass substrate and then dried at room temperature and ∼70% relative humidity under natural convection. After the water was completely evaporated, the sample was soaked in toluene (99.8%; Sigma-Aldrich) for 2 h, and then rinsed with toluene to completely eliminate oil residuals. Finally, the PVA membrane was dried under a vent hood, and can then be gently peeled off from the glass substrate. The peel-off was performed with a tweezer directly in the air before crosslinking or under acetone after crosslinking the PVA with GA.

### Crosslinking PVA membrane with GA

To prepare the water-resistant surface, the PVA membrane was crosslinked with GA via a solution-phase method^51^ after fabrication. For the crosslinking reaction, the dry PVA membrane was immersed in 10 vol% GA (50 wt% in water; Sigma-Aldrich) and 90 vol% acetone (≥99.8%; Merck) at 40 °C for 24 h. Hydrochloric acid (HCl, 37 wt%; Sigma-Aldrich) was added to adjust the pH 2–3. After the reaction, the membrane was washed several times with distilled water and then immersed in distilled water for 24 h at room temperature to eliminate any possible residuals of HCl and GA. The crosslinked PVA was finally dried under a ventilated hood.

### Contact angle measurement

The contact angle was measured with custom-built instrumentation. The test surface was put on a tilting stage (Sigma Koki, GOH-40A35) mounted on an elevating stage to control the levelness and height of the surface. The stage system was placed in between a light source and a CCD (charge-coupled device) camera (high-speed camera; MotionPro X4, IDT, Taiwan, and Phantom M110) equipped with a lens (Sigma, 30 mm, f/1.4). To measure the static contact angle, a sessile drop (3–5 μl) was dispensed onto the test surface with a pipette (DragonLab, 0.5–10 μl), and a side-looking image was captured with the camera when the droplet was stabilized. To measure the advancing contact angle, a 3 μl drop was created on the surface and held with a syringe (Terumo Syringe, 1 cc ml^−1^). The liquid was then pumped into the drop through a needle until the drop volume increased to 10–20 μl. The pumping speed was 0.1 μl s^−1^ and was controlled with a high-precision syringe pump (Longer Pump, LSP01-2A). Inversely, when measuring the receding angle, the liquid was pumped out from the 10–20 μl drop at the same speed until the remaining liquid detached from or was distorted by the needle. The drop expansion and contraction processes were captured with the CCD camera at ten frames per second. Both static and dynamic contact angles were calculated from the videos by DropSnake in ImageJ.

### Hydrostatic pressure manipulation

A custom-made setup was used to manipulate the hydrostatic pressure in the measurement of breakthrough pressure as well as visualization of the reversible Cassie–Wenzel transition (Supplementary Fig. 16). The test surface was placed in a sealed glass chamber filled with water (the working liquid) and observed under an optical microscope (Nikon Eclipse TS100) equipped with a high-speed camera (Phantom M110). The chamber was connected to a syringe (Terumo Syringe, 10 cc ml^−1^) that initially stored unpressurized air. The air was then compressed with a high-precision syringe pump (Longer Pump, LSP01-2A) at a volumetric flow rate of 100 ml h^−1^. In turn, the hydrostatic pressure of the water increased. The exact amount of pressure increase can be determined according to the variation in the air volume. Details for calculating the hydrostatic pressure are provided in the Supplementary Note 5.

### Preparation of PDMS micro-cavity and micro-bead surfaces

Both the PDMS micro-cavity and the micro-bead surfaces were fabricated via the replica moulding technique using the same micro-cavity mould. The mould was made of PVA using MET technique. The PDMS micro-cavity surface was produced in a two-step replica moulding process. First, the polystyrene (PS) precursor solution (dissolved in toluene) was cast onto the PVA mould. After toluene evaporation, the PS-negative replica with micro-bead structure was obtained by dissolving the PVA mould in water. In the second replica moulding process, the PDMS precursor containing 10 wt% curing agent (Sylgard 184 silicone elastomer kit, Dow Corning) was cast onto the PS-negative replica and cured at 80 °C for 2 h. Subsequently, the crosslinked PDMS micro-cavity surface was prepared by dissolving the PS mould in toluene. During fabrication of the PDMS micro-bead surface, the PDMS precursor containing 10 wt% curing agent was directly casted onto the PVA mould. This was crosslinked at 80 °C for 2 h followed by dissolving the PVA mould in water. Here, dissolving the mould preserves the integrity of the re-entrant structure for both micro-cavity and micro-bead surfaces, better than peeling the replica off from the mould.

### Mechanical stability test

The surfaces' mechanical stability was characterized with the sandpaper abrasion test^21^. The surface was placed down on the sandpaper (400 Cw) and forced to move along the ruler for 10 cm at 0.5 cm s^−1^. The surface was then rotated 90° and moved for another 10 cm at the same speed facing down on the sandpaper. This is one cycle of the abrasion test. To further increase the friction between the surface and sandpaper, the test surface was loaded with increasing amount of weight. For every load, one cycle abrasion test was carried out after which the water contact angle was measured. The applied pressure on the surface was calculated by dividing the load by the surface area.

### Stretching test

The stretching test was conducted with a custom-built setup. First, a strip of the PVA porous membrane had its two ends glued correspondingly onto two glass substrates. The glass substrates were then pulled by two high high-precision syringe pumps (Longer Pump, LSP01-2A) after attached to the syringe pumps by adhesive tapes. The rate of the stretch was controlled by the syringe pumps at a constant speed of 400 μm s^−1^ (see Fig. 5c and Supplementary Movie 5).

### Chemical stability test

Two tests of chemical stability were performed. In the first test, the apparent contact angle of water droplets was measured on the crosslinked PVA omniphobic surface at different pH values. HCl and NaOH were used to tune the water droplet into acid and alkali, respectively. In the second test, the crosslinked PVA omniphobic surfaces were immersed in 1 M HCl and 1 M NaOH solutions, respectively. The apparent contact angle of water droplet was measured every 24 h for HCl immersion and 2 h for NaOH immersion, respectively.

### Data availability

The data that support the findings of this study are available from the corresponding author on request.

## Additional information

**How to cite this article:** Zhu, P. *et al*. Well-defined porous membranes for robust omniphobic surfaces via microfluidic emulsion templating. *Nat. Commun.* **8**, 15823 doi: 10.1038/ncomms15823 (2017).

**Publisher's note:** Springer Nature remains neutral with regard to jurisdictional claims in published maps and institutional affiliations.

## Supplementary Material

Supplementary Information Supplementary Figures, Supplementary Table and Supplementary Notes

Supplementary Movie 1 The sustained Cassie state during a water droplet evaporation. No infusion of water into the micro-cavity is observed at any time instant during the droplet evaporation, indicating the persistence of the Cassie state. The video is played at a 50x speed (compared to real time).

Supplementary Movie 2 The sustained Cassie state during a dimethyl carbonate (DMC) droplet evaporation. During the evaporation of DMC, most micro-cavities are in the Cassie state, except that several sparsely distributed ones are wetted, as displayed by the white regions. The video is played at a 10x speed.

Supplementary Movie 3 Flushing away the ink residual. The ink stain on the porous membrane is readily flushed away by distilled water, demonstrating that the residual is initially situated on top of the surface and the surface is self-cleaning. The video is played in real time.

Supplementary Movie 4 Reversible Cassie-Wenzel wetting transition. Initially, the liquid (water) is in the Cassie state. Then the Cassie state transitions to the Wenzel state when the pressure is gradually elevated. In the Wenzel wetting state, it is observed that every micro-cavity is in a Janus state: the white part indicates water invasion, while the black part denotes the compressed air pocket. Then, the Cassie state recovers when the pressure is decreased. The video is played at a 2x speed.

Supplementary Movie 5 Unidirectional stretching of the flexible porous membrane. The video is played in real time.

Supplementary Movie 6 Deformation of the micro-cavity under unidirectional stretching. With an increase in the stretching strain, the micro-cavity gradually deforms from a pancake-like shape into a prism-like shape. The video is played at a 0.5x speed.
