# Supplementary material for: Well-defined porous membranes for robust omniphobic surfaces via microfluidic emulsion templating
Source: Nat Commun. 2017 Jun 12;8:15823. doi: 10.1038/ncomms15823 (PMC5472779; doi:10.1038/ncomms15823)
Supplement: Supplementary Information — Supplementary Figures, Supplementary Table and Supplementary Notes [file ncomms15823-s1.pdf]

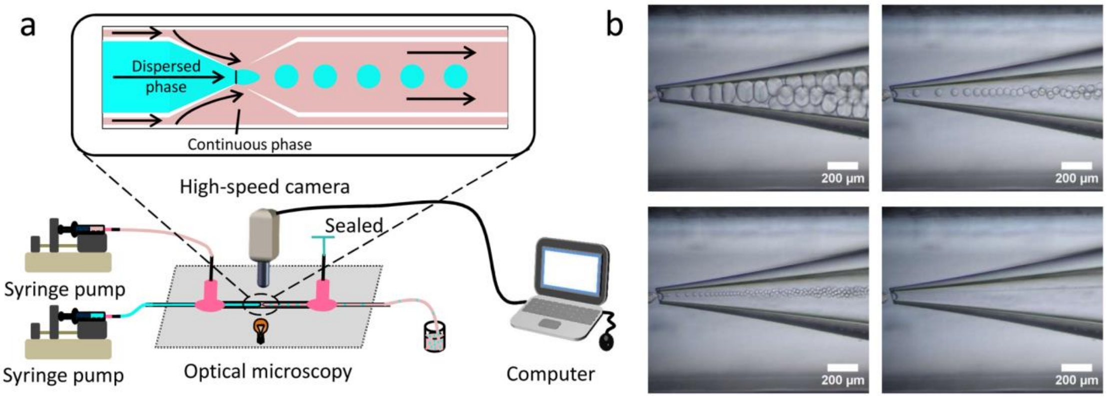


**Supplementary Figure 1. Production of uniform microemulsions in capillary microfluidic devices.** (**a**) Schematic of the experimental setup. The dispersed and continuous phase fluids are supplied by two syringes, with the volumetric flow rate precisely controlled by syringe pumps. The process of droplet generation is observed under an optical microscope, recorded by a high-speed CCD camera, and visualized by a computer. (**b**) Droplet size tuned by flow rates. The dispersed phase flow rate is fixed at 0.1 mL $h^{-1}$, while the continuous phase flow rates are 0.025 mL $h^{-1}$, 0.65 mL $h^{-1}$, 1.5 mL $h^{-1}$, and 8 mL $h^{-1}$ for images from left to right, up to bottom, and the corresponding droplet sizes are 117.4 μm, 48.8 μm, 27.4 μm, and 3.6 μm in diameter. The droplets are highly uniform.

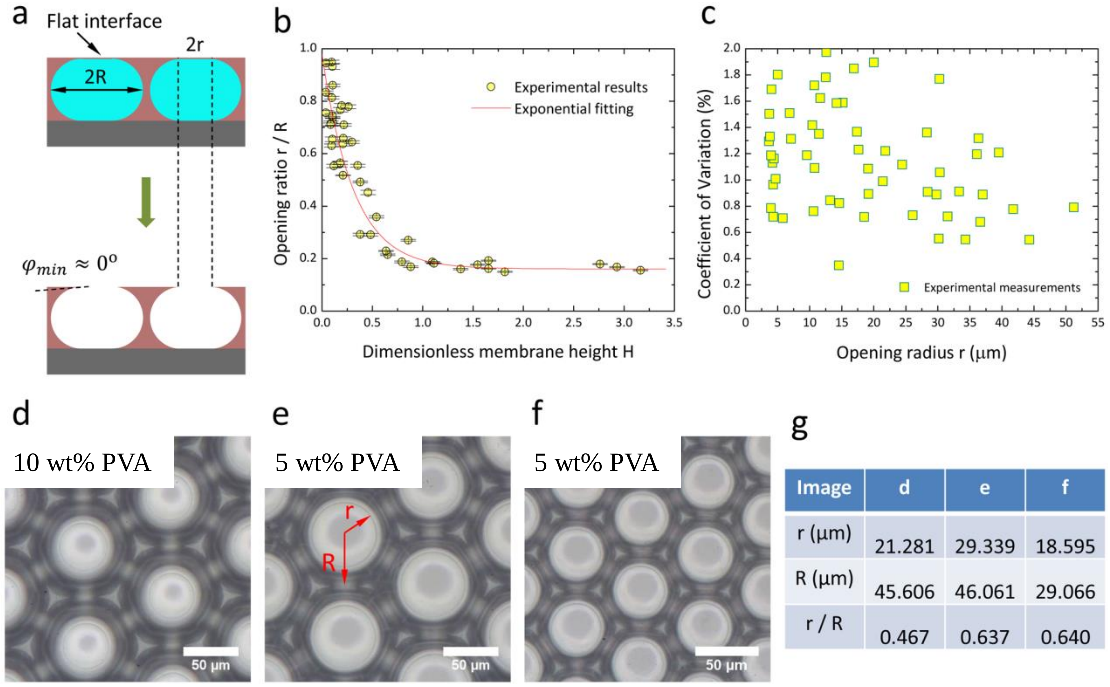


| Image | d | e | f |
|---|---|---|---|
| r (μm) | 21.281 | 29.339 | 18.595 |
| R (μm) | 45.606 | 46.061 | 29.066 |
| r / R | 0.467 | 0.637 | 0.640 |

**Supplementary Figure 2. Precise control of the porous structure.** (**a**) Creating of narrow openings in the step of template removal. The top flat interface (upper schematic) of the pancake-like droplet is ultrathin, and eliminated for the formation of opening (lower schematic) with radius *r*. As a result of the natural deformation of the droplet template and the elimination of the flat interface, the opening angle $\varphi_{mnin}$ is around 0 °. (**b**) Opening ratio *r/R* controlled by the dimensionless membrane height *H* = $c_{PVA}V_{PVA}\rho_{solution}$ / ($\rho_{solid}SR$), where $c_{PVA}$, $V_{PVA}$, $\rho_{solution}$, $\rho_{solid}$, *S*, and *R* are the PVA solution concentration, deposit volume of the PVA solution, density of the PVA solution, density of the solid PVA, deposit area of the emulsion, and radius of the droplet template, respectively. A smaller *H* indicates a larger deformation of the droplet template, thereby a larger opening ratio. Various droplet sizes are tested in (**b**), with the value of *R* ranging between 23 μm and 53 μm. (**c**) Coefficient variation (CV) of the narrow opening radius. For all *r* tested in the range of 3.7 μm to 51.2 μm, CV is less than 2%, indicating that the opening is highly uniform. Ten measurements were performed to determine the CV value for each opening radius *r*. (**d**-**f**) Optical micrographs showing PVA porous membranes with different values of *r* and *R*. (**d**) and (**e**) are produced by using droplet templates with the same size, but at different PVA concentrations (different *H*): 10 wt% PVA for (**d**) and 5 wt% PVA for (**e**). (**e**) and (**f**) are produced by using droplet templates with different sizes, but at the same concentration of PVA (5 wt%) and the same value of *H*. (**g**) Measurements of *r* and *R* for micrographs of (**d** to **f**). (**d**) and (**e**) are the same in *R*, but quite different in *r*; (**e**) and (**f**) are the same in *r/R*, but different in *r* and *R*. Therefore, the ratio *r/R* and opening radius *r* can be manipulated independently, where *r/R* is controlled by dimensionless height *H*, as shown in (**b**), and *r* is controlled by varying droplet radius *R* once the ratio *r/R* is determined. In principle, *r/R* can be designed large by using dilute PVA solution (small *H*), and the opening radius *r* can be simultaneously made small by choosing droplet template of a small radius *R*.

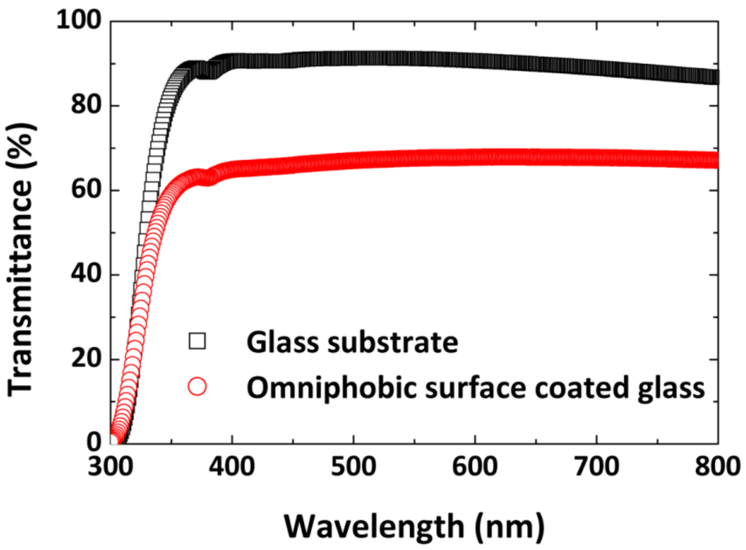


**Supplementary Figure 3. Ultraviolet-visible transmittance spectra of a glass slide and a PVA porous membrane coated glass.** The solid fraction of the test omniphobic surface is $f_s$ = 21%. For light wavelength in the visible spectra ranging from 380 to 780 nm, the transparency of the omniphobic surface coated glass is reduced by ~20% compared to that of the bare glass substrate. The reduction in transparency results from the absorption of light by PVA (with the thickness ranging from tens to hundreds of micrometers) and reflection of light on the curved air-PVA interface on the micro-cavities. We anticipate that higher light transmittance is possible by further reducing the solid fraction of the omniphobic surfaces.

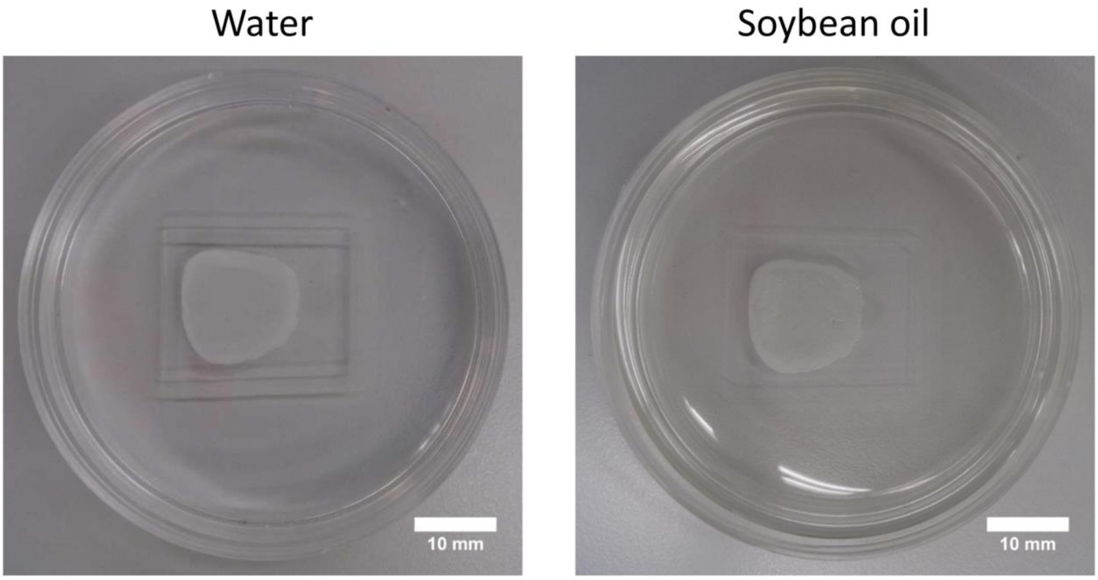


**Supplementary Figure 4. Immersion of the PVA porous membrane in water and soybean oil.** The shiny white part in the middle of the petri dish indicates the air pocket trapped inside the micro-cavity when the porous membrane is immersed in both water and soybean oil. The air cushion observed in the picture is similar to that identified in springtail immersion test.

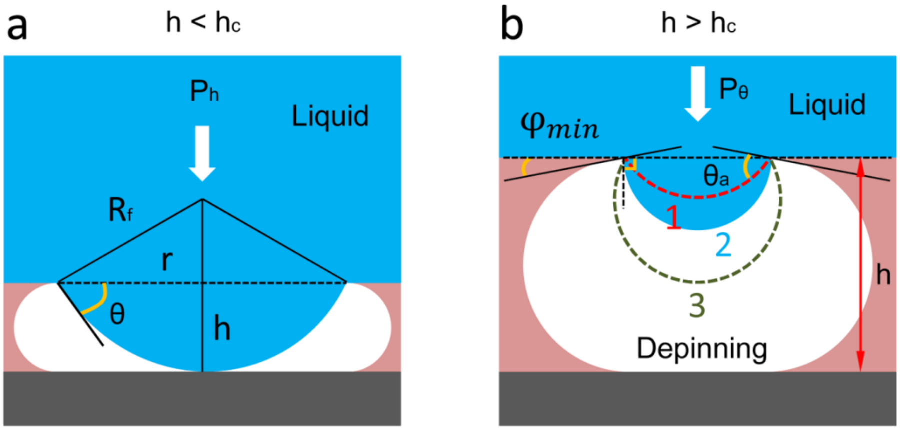


**Supplementary Figure 5. The breakthrough pressure in two sagging scenarios.** (**a**) Breakthrough pressure $P_h$ in the case that the liquid front contacts the bottom substrate. $r$ is the radius of the opening, $h$ is the height of the structure, and $R_f$ is the radius of the sagging interface. In this case, angle $\theta$ is essentially smaller than the advancing angle $\theta_a$. (**b**) Breakthrough pressure $P_\theta$ in the case of contact line depinning. Depending on the value of $\theta_a - \varphi_{mnin}$, three possibilities may occur for the depinning: case 1 (red dashed line) where $\theta_a - \varphi_{mnin} < 90°$, case 2 (blue solid interface) where $\theta_a - \varphi_{mnin} = 90°$, and case 3 (olive dashed line) where $\theta_a - \varphi_{mnin} > 90°$.

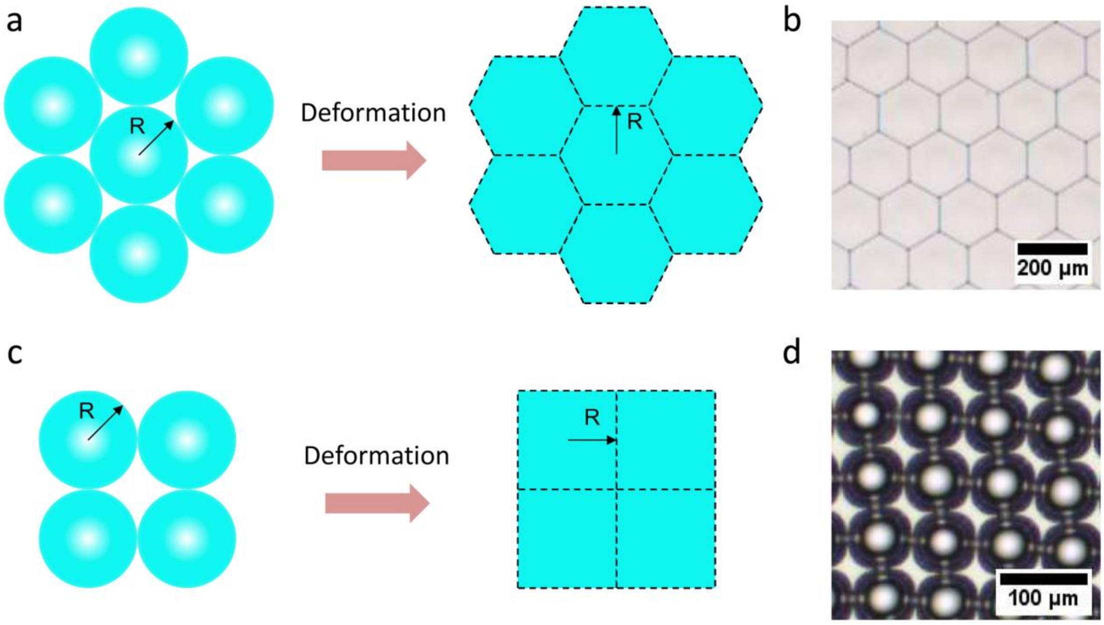


**Supplementary Figure 6. Deformation of droplet templates during solvent evaporation.** (**a**) Hexagonally arrayed droplets deform from spherical shape into hexagonal prisms of height *h*. (**b**) Optical image showing the deformation case in (**a**). (**c**) Schematic of rhombus-arrayed droplets deformation. (**d**) Optical image of a porous membrane with rhombus-arrayed micro-cavities.

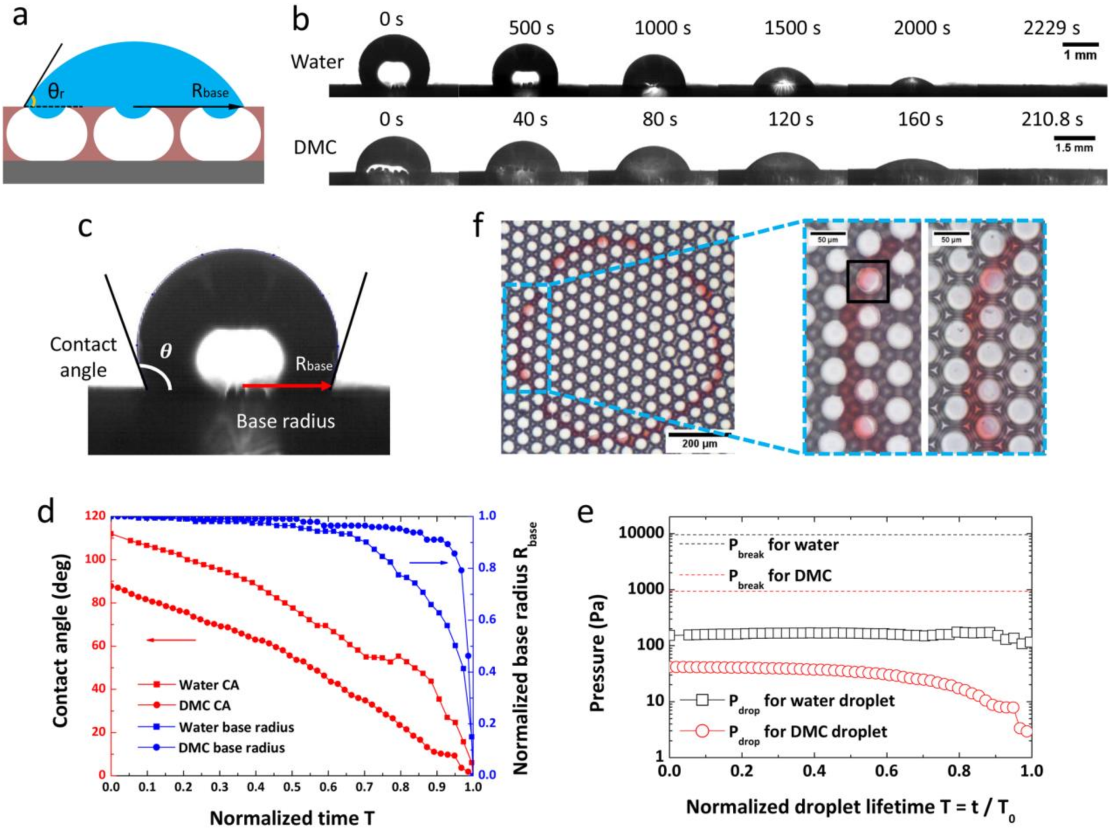


**Supplementary Figure 7. Evaporation of liquid drops on the porous omniphobic surface.** (**a**) Schematic of the cross-section profile of a droplet deposit during evaporation. $\theta_r$ is the receding angle of the liquid, and $R_{base}$ is the base radius of the liquid deposit. (**b**) Series of images displaying water and DMC droplets evaporation on the porous omniphobic surface. (**c**) Image of a droplet during evaporation. Both the apparent contact angle $\theta$ and base radius $R_{base}$ are time dependent. (**d**) The plot of apparent contact angle and normalized base radius versus normalized time for water and DMC evaporation. The base radius is normalized by the initial value when the droplet is deposited at time $t$ = 0. The time is normalized by droplet lifetime $T_0$, 2229 s for water and 210.8 s for DMC. (**e**) Variation of Laplace pressure $P_{drop}$ (symbol) during water and DMC droplets evaporation. $P_{drop}$ is always smaller than the breakthrough pressure $P_{break}$ (dashed line) for the entire lifetime of both water and DMC droplets. (**f**) A hexagonal pattern of an ink residual after water evaporation. The residual is situated atop the porous surface, as demonstrated in the right two magnifications, where the left one is focused on the top layer of the surface, while the right one is focused on the bottom. The edge of the residual pattern is sharp and clear in the left magnification (black box) but dim in the right one.

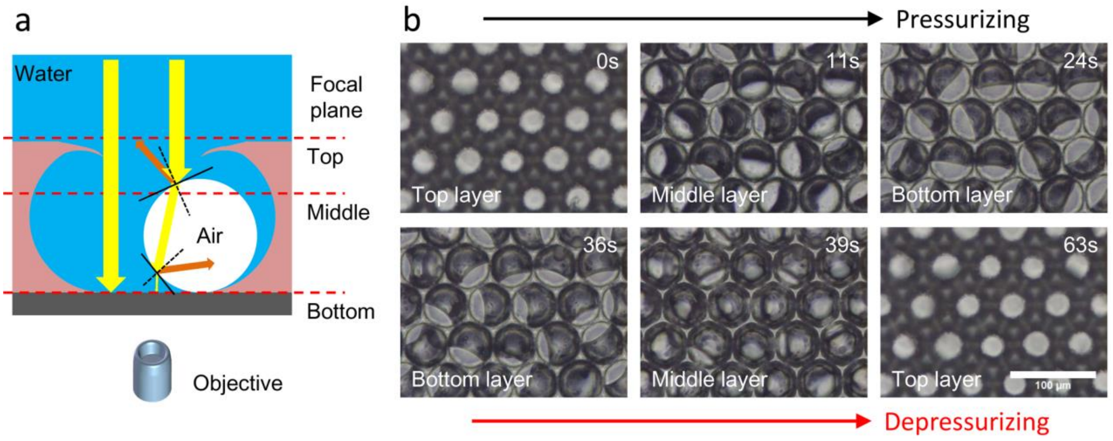


**Supplementary Figure 8. Observing the reversible wetting transition process.** (**a**) Schematic showing the light path under an inverted transmitted light microscopy (not scale). The yellow arrow indicates the transmitted light and the orange arrow denotes reflection on the air-water interface. The width of the arrow represents the luminous intensity. The absorption of light by the medium is not displayed. Owing to the reflection and refraction of light on the air-water interface, the focal plane should be adjusted in the range between the top to the bottom layer for a clear visualization of the wetting process. (**b**) Snapshots of the reversible wetting process at different instants. At the pressurizing stage, the air is compressed so that the air-water interface lowers with the increasing pressure. In this case, the focal plane is correspondingly lowered. So at 0 s (top left image, before pressurizing), the focal plane is on the top layer, where the light part denotes the narrow opening of the micro-cavity, and the dark part represents the solid polymer structure. At 11 s (top middle image), the focal layer is lowered to the middle plane, where the light part is water phase and the dark part is the compressed air because of the reflection on the air-water interface. At 24 s (top right image), the focal plane is at the bottom layer of the membrane, where the circular boundary between micro-cavities is clear. In this case, the light part still stands for water and the dark part for air. In the process of depressurizing, the focal plane is tuned inversely from the bottom to the top layer. Finally, at 63 s, the system restores the non-wetting state, and the light circle indicates the top narrow opening.

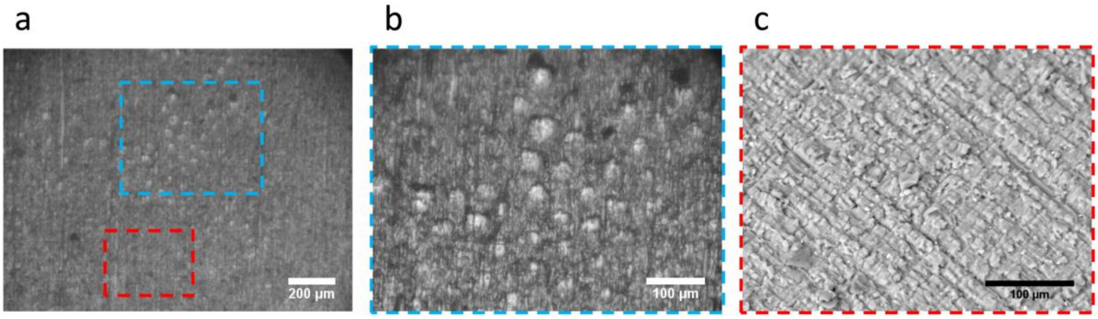


**Supplementary Figure 9. Surface morphology of PDMS micro-cavity structures after 100 cycle abrasions.** (**a**) Optical image of the surface morphology. Surface structures are almost wiped out (dashed red box), except a small portion of the bottom micro-cavity left (dashed blue box). (**b**) Optical micrograph showing the magnification of the preserved bottom structures. (**c**) SEM image showing the surface roughness of the structure damaged area.

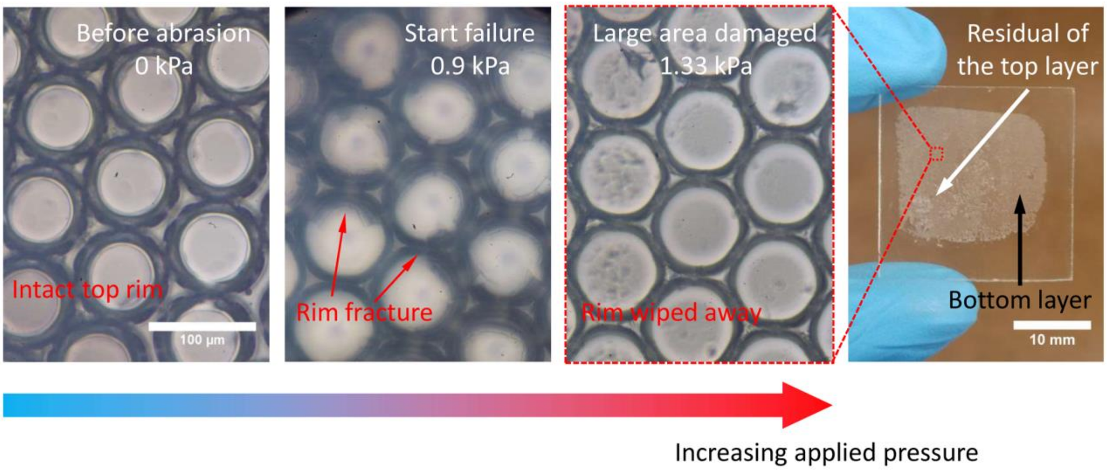


**Supplementary Figure 10. Structure failure behavior of the PVA porous membrane with increasing applied pressure in sandpaper abrasion test.** Before the test, the rim of the narrow opening on the top layer is intact (left first image). At the pressure of 0.9 kPa, the top rim is fractured (left second image). At a higher pressure of 1.33 kPa, the top layer is almost wiped away but the bottom structures still maintain (right second image). The rightmost picture displays the sample after being abraded at the pressure of 1.33 kPa, where the bottom structure is still firmly attached to the substrate and small amount of the top layer residual is observed. For this sample, the size is $R$ = 44.7 µm, $r/R \approx 0.63$.

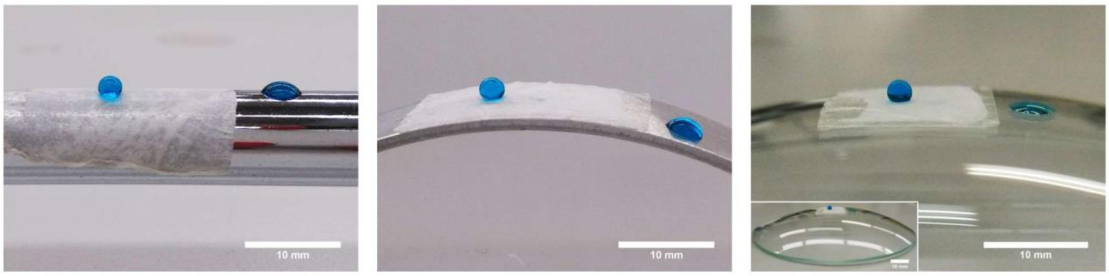


**Supplementary Figure 11. Examples of the flexible omniphobic surface transferred onto various materials with different shapes using double-faced adhesive tape.** The substrates are cylindrical stainless steel, aluminum curved beam, and glass spherical cap respectively from left to right. For all cases, the water droplet (dyed with methylene blue) beads up on the coating while wets the naked parts.

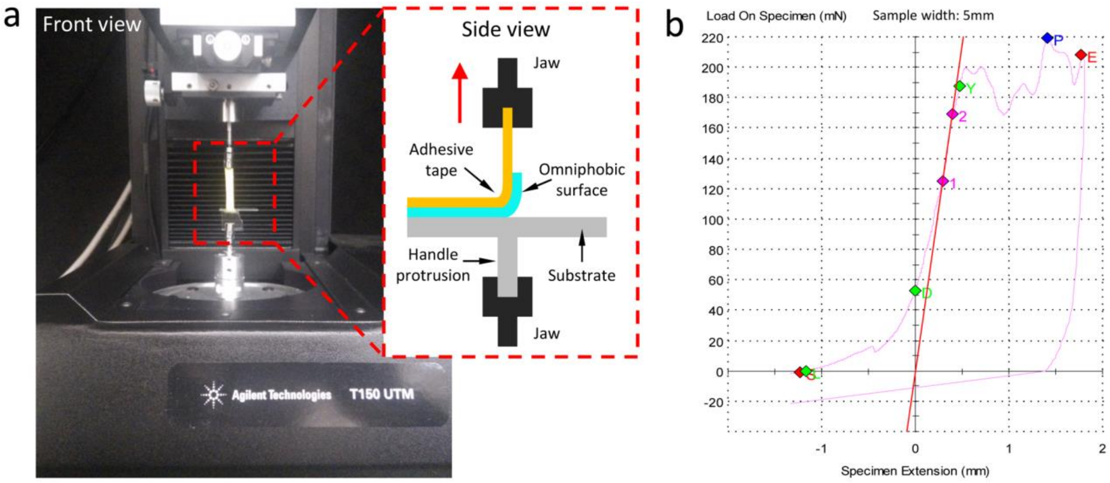


**Supplementary Figure 12. Measurement of the adhesion force between the omniphobic coating and the substrate.** (**a**) Experimental setup for the measurement of peel adhesion. The adhesion force is measured by a micro-tensile tester (Agilent, T150 UTM), as shown in the front view picture. To eliminate the influence of the deformation of the porous membrane when stretching, a strip of adhesive tape is firmly attached to the omniphobic coating before the measurement. Then the adhesive tape side is clamped into the upper jaw of the tensile tester. The substrate is placed horizontally and secured to the lower jaw by clamping the handle protrusion firmly (the handle protrusion is glued perpendicularly to the substrate if necessary), as shown in the schematic of the side view. After positioning the sample, the coating is peeled by the two jaws at an angle of 90° at a constant speed of 20 μm $s^{-1}$. (**b**) The measurement of adhesion force for one omniphobic sample with the width of 5 mm. At the initial stage of peeling, the adhesive tape is at relaxation until reaching the point “Y”. After that, the coating starts to be peeled off from the substrate (in the extension range between point “Y” and “E”) from which the adhesion force varies between ~170 and ~220 mN, with an average value about 200 mN. The adhesion can then be obtained by dividing the tensile force with the width of the sample, about 40N $m^{-1}$ between the coating and the glass substrate.

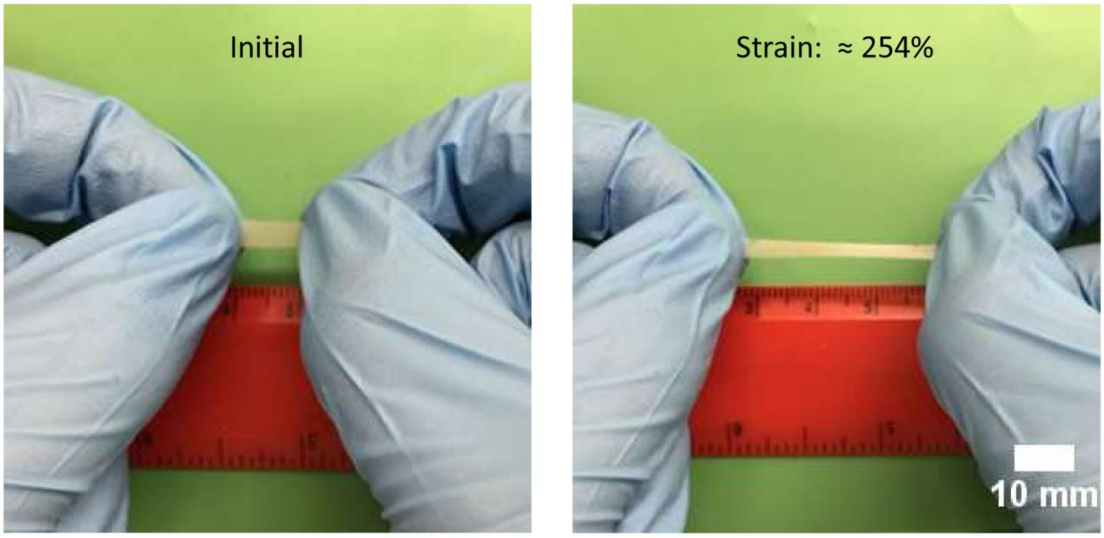


**Supplementary Figure 13. Stretching the flexible porous membrane.** The tensile strain can be as large as 254%.

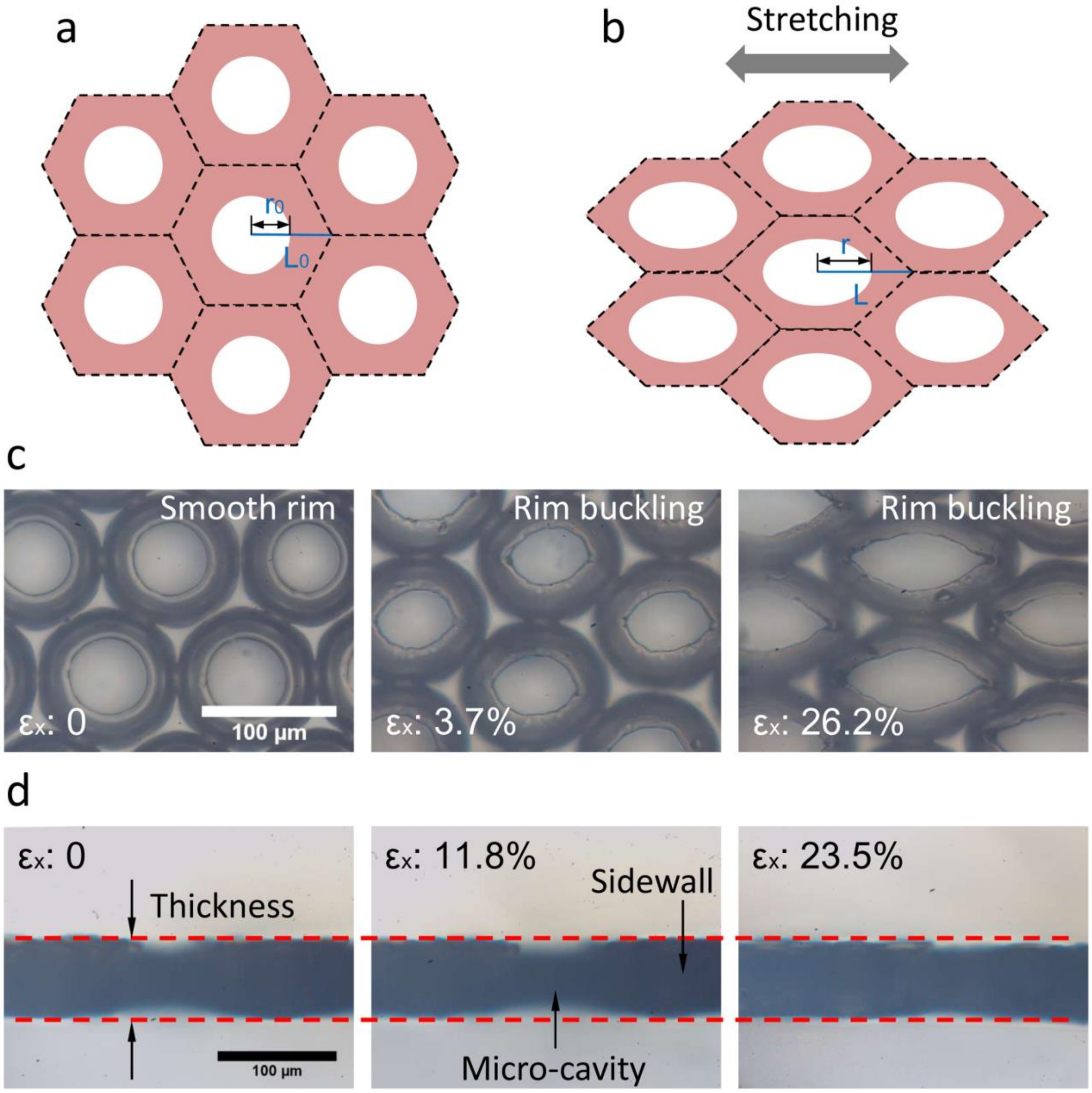


**Supplementary Figure 14. Deformation of the porous membrane.** (**a**) Schematic of the top view of the un-stretched surface. $r_0$ is the initial radius of the opening, and $L_0$ is the initial characteristic length of the hexagonal cell. (**b**) Schematic of the top view of the unidirectional-stretched surface. $r_0$ and $L_0$ change into $r$ and $L$, respectively. Note that $r$ and $L$ are anisotropic along different directions, because of the anisotropic strain. (**c**) Buckling of the rim of the narrow opening under tensile strain. The buckling induces volume variation of the micro-cavity, by which the height of the sidewall can be invariant at strains of $\varepsilon_x < 30\%$. (**d**) Optical microscopic images showing the almost constant thickness of the membrane at tensile strains of $\varepsilon_x < 30\%$.

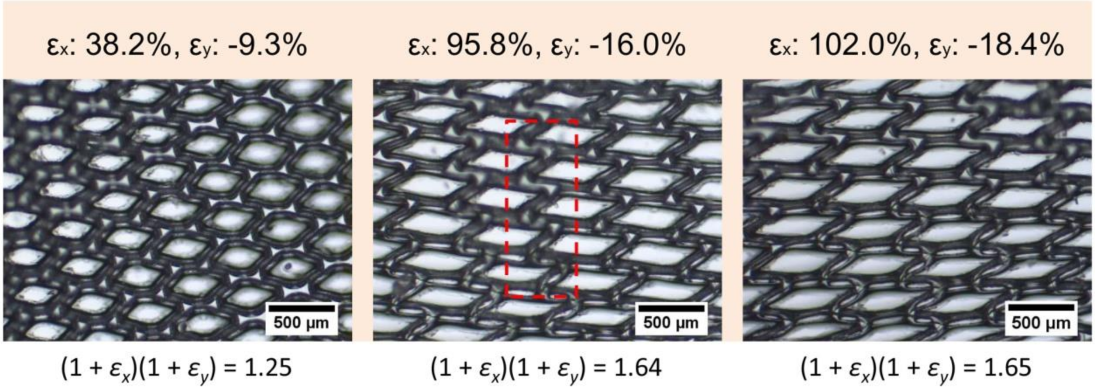


**Supplementary Figure 15. Plastic deformation of the porous membrane at large strains.** In this case, the solid material is deformed by the imposed strains, as indicated by the dashed red box where buckling of the sidewall is observed. The deformation of the solid material is also manifested in the relation of $(1 + \varepsilon_x)(1 + \varepsilon_y) > 1$, and the product of $(1 + \varepsilon_x)$ and $(1 + \varepsilon_y)$ increases with the strain. This type of deformation is, therefore, irreversible.

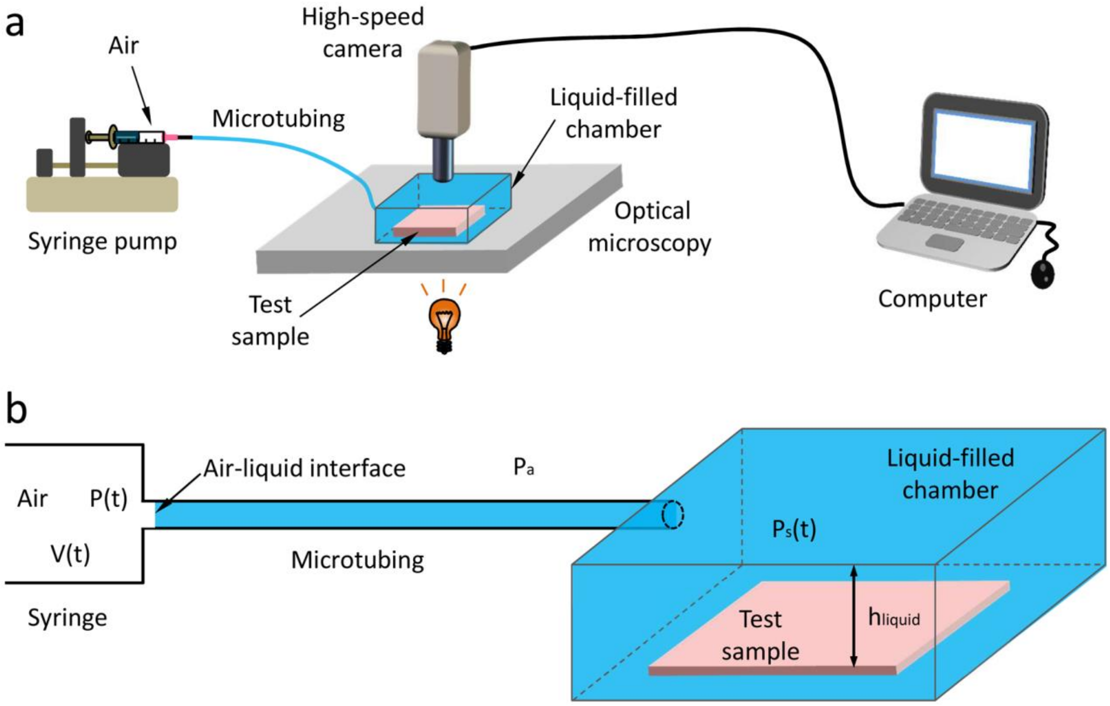


**Supplementary Figure 16. Liquid immersion test.** (**a**) Experimental setup for the liquid immersion test. The sample is placed in a sealed chamber that is pre-filled with the test liquid. A syringe stored a specific volume of air is connected to the chamber by a microtubing. The pressure of the liquid is elevated by compressing the air stored in the syringe using a syringe pump. The whole process of the immersion test is observed under an inverted optical microscope, and recorded by a high-speed CCD camera that is connected to a computer. (**b**) Schematic of the detailed connection between the syringe and liquid-filled chamber.

**Supplementary Table 1. Material property of the test liquids**

| Liquid | Surface tension (mN / m) | $\theta_Y$ (°) |
|---|---|---|
| Water | 72.8 | 71.7 |
| Glycerol | 64 | 68.4 |
| 2% SDS | 36.5 | 15.1 |
| 1,4-dioxane | 33 | 8.6 |
| Olive oil | 32 | 25.9 |
| Soybean oil | 29.43 | 17.2 |
| DMC | 28.5 | 9.5 |
| 2-octanol | 27.6 | 10.0 |
| Hexadecane | 27.1 | 15.1 |
| Paraffin oil | 26 | 15.9 |

**Supplementary Note 1: Determining the breakthrough pressure**

The Cassie-Wenzel transition occurs when the pressure difference across the liquid-vapor interface exceeds a critical value, denoted as the breakthrough pressure $P_{break}$. The wetting transition is induced by either the liquid front making contact with the substrate (Supplementary Fig. 5a) or depinning of the three-phase contact line (Supplementary Fig. 5b).

In the scenario of Supplementary Fig. 5a, $P_{break} = P_h = 2\gamma/R_f$, where $R_f$ is the radius of the liquid front, and $\gamma$ is the liquid surface tension. To determine $R_f$, we have the following geometric relationship (Supplementary Fig. 5a):

$$R_f \sin\theta = r , \tag{1}$$

$$R_f (1-\cos\theta) = h , \tag{2}$$

According to Supplementary Equations (1) and (2), $R_f$ is determined to be $R_f = (h^2 + r^2)/2h$. As such, the breakthrough pressure is calculated to be

$$P_{break} = P_h = \frac{4h\gamma}{h^2 + r^2} \text{ for } h < h_c . \tag{3}$$

In the scenario of Supplementary Fig. 5b, $R_f$ is determined by the advancing angle of the liquid $\theta_a$. Here, we consider arbitrary minimum geometric angle $\varphi_{min}$, and $R_f$ is expressed of the form $R_f = r/\sin(\theta_a - \varphi_{min})$. The minimum value of $R_f$ is $r$ when $\theta_a - \varphi_{min} \geq 90°$. The breakthrough pressure is determined by the minimum $R_f$ during the wetting transition. In case 1 (shown in Supplementary Fig. 5b) where $\theta_a - \varphi_{min} < 90°$, the breakthrough pressure is simply written as $P_{break} = P_\theta = 2\gamma/R_f = 2\gamma\sin(\theta_a - \varphi_{min})/r$. The critical membrane height $h_c$ is the depth of the liquid front, $h_c = R_f(1 - \cos(\theta_a - \varphi_{min})) = r(1 - \cos(\theta_a - \varphi_{min}))/\sin(\theta_a - \varphi_{min}) = r\sin(\theta_a - \varphi_{min})/(1 + \cos(\theta_a - \varphi_{min}))$. In cases 2 and 3, where $\theta_a - \varphi_{min} \geq 90°$, the breakthrough pressure is determined by the moment when $R_f = r$. As such, $P_{break} = P_\theta = 2\gamma/R_f = 2\gamma/r$. Now, the critical membrane height $h_c$ is of the form $h_c = r$.

In summary, we determine the breakthrough pressure $P_{break}$ depending on the relative difference between $\theta_a - \varphi_{min}$ and 90° as follows:

$$\text{for } \theta_a < 90° + \varphi_{min}, \quad P_{break} = \begin{cases} P_\theta = \dfrac{2\gamma\sin(\theta_a - \varphi_{\min})}{r}, h > \dfrac{r\sin(\theta_a - \varphi_{\min})}{1+\cos(\theta_a - \varphi_{\min})} = h_c \\ P_h = \dfrac{4h\gamma}{h^2 + r^2}, h < h_c \end{cases} ; \tag{4}$$

$$\text{for } \theta_a \geq 90° + \varphi_{min}, \quad P_{break} = \begin{cases} P_\theta = \dfrac{2\gamma}{r}, h > r = h_c \\ P_h = \dfrac{4h\gamma}{h^2 + r^2}, h < h_c \end{cases} . \tag{5}$$

**Supplementary Note 2: Height of the porous membrane**

The height of the porous membrane is determined by the deformation of droplet templates. For most cases, droplets self-assemble into dense-packed hexagonal arrays, and deform into pancake-like shape during membrane fabrication. Considering the extreme case that all spherical droplets of radius $R$ deform into prisms with hexagonal cross-section and height $h$, as shown in Supplementary Figs. 6a and 6b. Applying conservation of the droplet volume, we have the formula, $\frac{4}{3}\pi R^3 = 2\sqrt{3}R^2 h$. We then obtain $h = 2\sqrt{3}\pi R/9$ that sets a lower limit for the height of the membrane. Therefore, the height of the porous membrane fabricated by the MET method inherently has

$$h > \frac{2\sqrt{3}}{9}\pi R > R. \tag{6}$$

In very limited cases, we observe that droplets are arrayed in a rhombus pattern, as shown in Supplementary Figs. 6c and 6d. Adopting a similar volume conservation calculation, we have $\frac{4}{3}\pi R^3 = 4R^2 h$, and $h = \pi R/3$ as the lower limit of the membrane height. Therefore, for rhombus droplet arrays, the height of the membrane gives

$$h > \frac{\pi}{3} R > R. \tag{7}$$

We see that in any of the two cases, the membrane height is larger than the radius of the spherical droplet, thereby larger than the critical height $h_c$ ($h > R > r > h_c$).

**Supplementary Note 3: Persistence of the Cassie state during droplet evaporation**

The robustness of the micro-cavity structure (high $P_{break}$) is also manifested in the persistence of the Cassie state during droplet evaporation. During droplet evaporation, the elevated Laplace pressure is a result of the shrinkage of the droplet radius $R_{drop}$. Basically, $R_{drop}$ is related to the base radius of the droplet $R_{base}$ in the form of $R_{drop} = R_{base}/\sin\theta_r$, where $\theta_r$ is the receding angle of the liquid (Supplementary Fig. 7a). The Laplace pressure is then determined to be $P_{drop} = 2\gamma/R_{drop} = 2\gamma\sin\theta_r/R_{base}$. Since $R_{base}$ is essentially larger than the radius of the opening $r$, $P_{drop} \leq 2\gamma\sin\theta_r/r$. To preserve the Cassie state, the condition of $P_{drop} < P_{break}$ should be met. By comparing $P_{drop} \leq 2\gamma\sin\theta_r/r$ and $P_{break}$ given in Supplementary Equations (4) and (5), we have the following condition for the persistence of the Cassie state:

$$\text{for } h > h_c, \qquad \theta_r < \min[\theta_a - \varphi_{min}, 90°]; \tag{8}$$

$$\text{for } h < h_c, \qquad \theta_r < \arcsin(\frac{2hr}{h^2 + r^2}). \tag{9}$$

Because $h > h_c$ always holds for micro-cavity structures, condition Supplementary Equation (8) determines the persistence of the Cassie state for the porous omniphobic surface in the present study.

Considering that $\varphi_{min} \leq 0°$ for any liquid deposits, we anticipate that droplet sustains the Cassie state throughout the evaporation process owing to $\theta_r < \theta_Y < 90°$ and $\theta_r < \theta_a < \theta_a - \varphi_{min}$ for all the test liquids. To confirm this, we examined, for example, water and DMC droplets evaporating on the PVA omniphobic surface (Supplementary Fig. 7b). After extracting the transient contact angle $\theta$ and base radius $R_{base}$ (Supplementary Figs. 7c and 7d), the calculated $P_{drop}$ ($R_{drop} = R_{base}/\sin\theta$) is found to be always smaller than $P_{break}$ for both liquids (Supplementary Fig. 7e), indicating a sustained Cassie state for the entire droplet lifetime. Furthermore, by filming the evaporation process under an optical microscope, we have confirmed this speculation in the main context (Fig. 3d and 3e), except that several cavities are sparsely wetted by DMC (Fig. 3e), probably due to defects at the rims of the corresponding openings (resulting in $\varphi_{min} > 0°$). It is remarkable to note that, in contrast to pillar-like arrays where sideways spreading of liquids may occur along with the wetting transition, the wetted micro-cavities are solitary and prevented from propagating by the impermeable sidewalls. As maintained in the Cassie state, the residual of a solution would sit atop the porous membrane after solvent evaporation, as shown in Supplementary Fig. 7f (see also Supplementary Movie 3). The hexagonal shape of the ring-like pattern, as a result of the hexagonal-arrayed openings, may offer new opportunities for liquid-based printing and biosensing applications.

**Supplementary Note 4: Apparent contact angle on deformed omniphobic surfaces**

In the Cassie state, the apparent contact angle is predicted by the Cassie-Baxter model. We therefore have

$$\cos\theta_{un}^{*} = f_{s-un}(\cos\theta_Y + 1) - 1, \tag{10}$$

$$\cos\theta_{def}^{*} = f_{s-def}(\cos\theta_Y + 1) - 1. \tag{11}$$

Replacing $\cos\theta_Y$ in Supplementary Equation (11) by using Supplementary Equation (10), we have the following relationship between $\theta_{def}^{*}$ and $\theta_{un}^{*}$:

$$\cos\theta_{def}^{*} = \frac{f_{s-def}}{f_{s-un}}(\cos\theta_{un}^{*} + 1) - 1. \tag{12}$$

In terms of surface deformation (Supplementary Fig. 14), we consider the one-dimensional solid fraction $f_s = (L - r)/L$ along the deformational direction, where $r$ is the radius of the opening, $L$ is the characteristic length scale of the hexagonal cell. By this definition, $f_{s\text{-}un} = (L_0 - r_0)/L_0$ and $f_{s\text{-}def} = (L - r)/L$, as shown in Supplementary Figs. 14a and 14b, respectively. For deformation at small strains, the surface deformation mainly comes from the deformation of the micro-cavity shape, and distortion of the solid material is negligible. To a first approximation, we have $L - r = L_0 - r_0$ to be a constant. As such, $f_{s\text{-}def} / f_{s\text{-}un} = [(L - r)/L]/[(L_0 - r_0)/L_0] = L_0/L$. Recalling the definition of strain $\varepsilon = (L - L_0)/L_0$, we therefore have $f_{s\text{-}def}/f_{s\text{-}un} = L_0/L = 1/(\varepsilon + 1)$ at small strains. Now, we determine the relationship between $\theta_{def}^{*}$ and $\theta_{un}^{*}$ in the stretching and compression direction respectively to be:

$$\cos\theta_{def}^{*} = \frac{1}{1+\varepsilon_x}(\cos\theta_{un}^{*} + 1) - 1; \tag{13}$$

$$\cos\theta_{def}^{*} = \frac{1}{1+\varepsilon_y}(\cos\theta_{un}^{*} + 1) - 1 = (1+\varepsilon_x)(\cos\theta_{un}^{*} + 1) - 1, \tag{14}$$

by applying $(1 + \varepsilon_x)(1 + \varepsilon_y) = 1$.

**Supplementary Note 5: Calculating the hydrostatic pressure for liquid immersion test**

The experimental setup for liquid immersion test is shown in Supplementary Fig. 16a. When air is compressed by the syringe pump, the pressure of the air $P$ elevates starting from the initial value of atmospheric pressure $P_a$. Since the air is compressed at a quite slow speed of 100 mL h$^{-1}$, the compression process is treated as an isothermal process. As such, we have the following simple relation to calculate to air pressure $P(t)$:

$$P(t) = P_a \frac{V_0}{V(t)}, \tag{15}$$

where $V_0$ is the initial volume of the air stored in the syringe, and $V(t)$ is the volume of air at time $t$. Compressing air at a constant speed, we have $V(t)$ in the form of

$$V(t) = V_0 - S_p t, \tag{16}$$

where $S_p$ is the compression speed, for example, $S_p$ = 100 mL h$^{-1}$. At a given time $t$, the hydrostatic pressure applied to the test omniphobic surface $P_s(t)$ is written as

$$P_s(t) = \rho g h_{liquid} + P(t) - P_a, \tag{17}$$

by neglecting the Laplace pressure jump across the air-liquid interface (Supplementary Fig. 16b). In Supplementary Equation (17), $\rho$ is the density of the liquid, $g$ is the gravitational acceleration, and $h_{liquid}$ is the height of the liquid above the test surface (the chamber is pre-filled with test liquid). The first term $\rho g h_{liquid}$ at the right-hand side of Supplementary Equation (17) is the contribution from the height of the liquid, and the second term $P(t) - P_a$ is the elevated pressure attributed to air compression. Combining Supplementary Equations (15)-(17), we have a time-dependent form of $P_s(t)$:

$$P_s(t) = \rho g h_{liquid} + P_a \frac{S_p t}{V_0 - S_p t}. \tag{18}$$

Using Supplementary Equation (18), we can readily determine the applied pressure at any time instant $t$ during air compression process.
